# Hypoxia-induced immortalization of primary cells depends on *Tfcp2L1* expression

**DOI:** 10.1038/s41419-024-06567-z

**Published:** 2024-02-28

**Authors:** D. Otero-Albiol, J. M. Santos-Pereira, A. Lucena-Cacace, C. Clemente-González, S. Muñoz-Galvan, Y. Yoshida, A. Carnero

**Affiliations:** 1 Instituto de Biomedicina de Sevilla, IBIS, Hospital Universitario Virgen del Rocío, Universidad de Sevilla, Consejo Superior de Investigaciones Científicas, Avda. Manuel Siurot s/n, 41013 Seville, Spain; 2 CIBER de CANCER, Instituto de Salud Carlos III (ISCIII), Madrid, Spain; 3 Centro Andaluz de Biología del Desarrollo (CABD), Consejo Superior de Investigaciones Científicas/Universidad Pablo de Olavide, 41013 Seville, Spain; 4 Department of Cell Growth and Differentiation, Center for iPS Cell Research and Application, Kyoto University, Sakyo-ku, Kyoto 606-8507 Japan

**Keywords:** Senescence, Mechanisms of disease

## Abstract

Cellular senescence is a stress response mechanism that induces proliferative arrest. Hypoxia can bypass senescence and extend the lifespan of primary cells, mainly by decreasing oxidative damage. However, how hypoxia promotes these effects prior to malignant transformation is unknown. Here we observed that the lifespan of mouse embryonic fibroblasts (MEFs) is increased when they are cultured in hypoxia by reducing the expression of *p16*^*INK4a*^, *p15*^*INK4b*^ and *p21*^*Cip1*^. We found that proliferating MEFs in hypoxia overexpress *Tfcp2l1*, which is a main regulator of pluripotency and self-renewal in embryonic stem cells, as well as stemness genes including *Oct3/4*, *Sox2* and *Nanog*. *Tfcp2l1* expression is lost during culture in normoxia, and its expression in hypoxia is regulated by Hif1α. Consistently, its overexpression in hypoxic levels increases the lifespan of MEFs and promotes the overexpression of stemness genes. ATAC-seq and Chip-seq experiments showed that Tfcp2l1 regulates genes that control proliferation and stemness such as *Sox2*, *Sox9, Jarid2 and Ezh2*. Additionally, *Tfcp2l1* can replicate the hypoxic effect of increasing cellular reprogramming. Altogether, our data suggest that the activation of *Tfcp2l1* by hypoxia contributes to immortalization prior to malignant transformation, facilitating tumorigenesis and dedifferentiation by regulating *Sox2*, *Sox9*, and *Jarid2*.

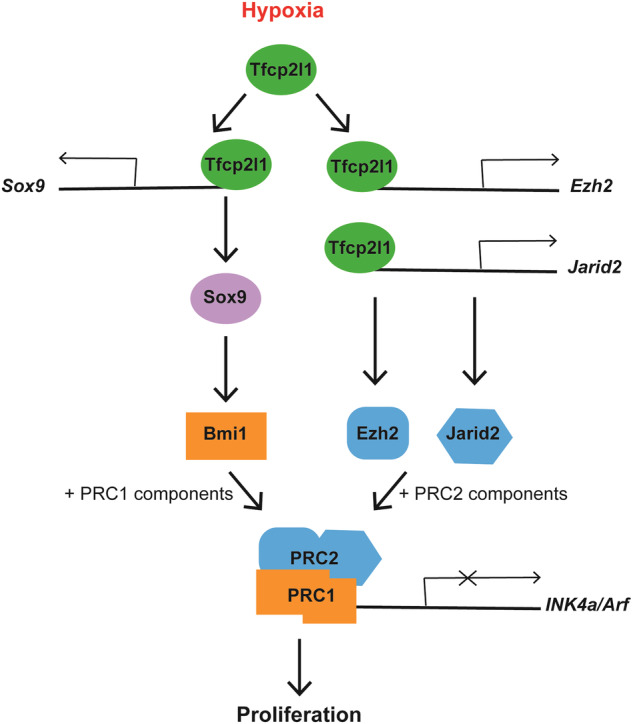

## Introduction

Senescence is a physiological state in response to stress and is characterized by irreversible proliferative arrest, changes in chromatin structure, specific epigenetic modifications, metabolic changes, high acid β-galactosidase activity, an increased secretion phenotype and organelle alterations [1]. It can be induced by intrinsic and extrinsic stimuli, such as telomere shortening, DNA-damage responses, oncogenic signaling detection, oxidative stress, and anti-cancer therapies [2]. It is also necessary for the regulation of different physiological processes, including tumor suppression [3, 4], the development of embryo structures, wound repair and cellular reprogramming in tissue repair [5]. Furthermore, cellular senescence correlates with tissue aging and is involved in the deleterious effects of age-related diseases [6].

Despite the number of physiological effects caused by cellular senescence, little is known about how low levels of oxygen, or hypoxia, which is a feature of every tissue in the organism [7], affect senescence and immortalization. Hypoxia can extend the lifespan of some primary cells [8–11] by decreasing oxidative damage [10]. However, how hypoxia promotes the evasion of senescence and whether this effect contributes to tumor malignancy is unclear [12, 13]. Additionally, hypoxia supports the pluripotency maintenance and self-renewal of embryonic stem cells (ESCs), induced pluripotent stem cells (iPSCs) and cancer stem cells (CSCs) through the activation of stemness genes such as *Oct3/4*, *Sox2* and *Nanog* [12–15]. For these reasons, here we investigated the effects of hypoxia on cellular senescence and immortalization and its possible relation to stemness physiology.

We show that hypoxia can extend the lifespan of MEFs through the activation of a main regulator of self-renewal and pluripotency, the transcription factor *Tfcp2l1*. According to our results, *Tfcp2l1* is transcriptionally regulated by Hif1α and binds to the regulatory regions of genes associated with proliferation or stemness, including *Sox2*, *Sox9*, *Jarid2* and *Ezh2*, as shown using ATAC-seq and ChIP-seq. Furthermore, we found that *Tfcp2l1* regulates the effect of hypoxia on cellular reprogramming. Our results suggest that the activation of *Tfcp2l1* by hypoxia could be relevant in immortalization prior to malignant transformation and the dedifferentiation, facilitating tumorigenesis and tumor malignancy.

## Methods

### MEFs isolation and culture

Wild-type MEFs were isolated from day 13.5 embryos derived from C57BL/6J mice. Briefly, pregnant females were sacrificed, and the whole uterus was extracted. Every embryo contained in the uterus was minced after removing red organs and the head. Then, tissue pieces were enzymatically dissociated using trypsin 2.5% (Invitrogen) and type IV collagenase (Merck). After this, MEFs were cultured under normoxic (20% O_2_) or hypoxic (3% O_2_) atmospheres with 5% CO_2_ at 37 °C. Hypoxia was achieved and maintained by flushing a hypoxic gas mixture in CO_2_ incubators (ThermoFisher Scientific). The culture medium used was DMEM supplemented with 10% fetal bovine serum (FBS; Life Technologies), penicillin 40 μg/mL, and streptomycin 40 μg/mL (Merck). The 3T3 modified protocol was performed after isolating the MEFs; cells were counted every 3 days, and 1×10^6^ cells were seeded in a new culture dish.

For treatments, 1 × 10^6^ cells were seeded, and, 24 h later, compounds were added to the culture medium: 6 mM dimetiloxalilglicina (DMOG) (Frontier Scientific) and 100, 500 or 1000 ƞg/μL doxycycline (Merck). After the indicated time for each experiment, the cells were fixed to assay acid β-galactosidase activity or collected for total RNA and total protein isolation.

All animal procedures were performed according to the experimental protocol approved by the IBIS and HUVR Institutional Animal Care and Use Committee (0309-N-15).

### Lentiviral vectors and cell transduction

HEK-293-T cells were used for the lentivirus production. Briefly, the cells were transfected with 1 mg/mL polyethylenimine, 10 μg lentiviral DNA plasmid, 8 μg psPAX2 plasmid vector coding *GAG* and *POL* genes and 2 μg pMD2G plasmid vector coding *VSV-G* gene. The HEK-293-T cells were then incubated in DMEM supplemented with lentiviral particles at 37 °C and collected 48 hours later. The lentivirus-containing medium was diluted 1:4 in DMEM and supplemented with 8 μg/mL polybrene (Merck). Finally, the medium was added to the target cells, which were incubated at 37 °C for 6 h. After 24 h, selection medium was added containing puromycin (Nucliber), blasticidin (Merck) or hygromycin B (Life Technologies).

The plasmids used to downregulate the expression of *Tfcp2l1* were kindly donated by Dr. Qi-Long Ying (University of Southern California): pLKO.1-Scramble-shRNA, pLKO.1-mTfcp2l1-shRNA#1 (GCAGGAATGTGAGGCCAAAGA) and pLKO.1-mTfcp2l1 shRNA#2 (GCTCTTCAATGCCATCAAAGG) [16]. pLenti-CMV-Tfcp2l1 was provided by Origene. The following plasmids were obtained from the Addgene repository: pLenti-CMV-MCS-GFP-SV (Addgene #73582), pLenti-CMV-rtTA3 (w756-1) (Addgene #26429), pcDNA3-mHif1α-Myc (P402A/P577A/N813A) (Addgene #44028), psPAX2 (Addgene #12260) and pMD2G (Addgene #12259). pLenti-CMVtight-Tfcp2l1 and pLenti-CMV- mHif1α-Myc (P402A/P577A/N813A) were provided by one of the authors. Finally, plasmids used for the iPSC generation are described elsewhere [17], and pMXs-*Tfcp2l1* was provided by one of the authors.

### iPSCs generation

The generation of miPSCs was performed as described previously [17], with some modifications: the oxygen concentration for hypoxia was 3% O_2_, the hypoxia treatment was 9 days long, *Tfcp2l1* cDNA was transduced using pMXs retroviral vector, and scrambled shRNA and shRNAs against *Tfcp2l1* mRNA were transduced using pLKO lentiviral vectors. Total RNA was isolated from MEFs 4 days after the transduction, and GFP^+^ colonies were counted 21 days after the transduction.

### Detection of acid β-galactosidase activity

Senescent and control MEFs were fixed in 0.5% glutaraldehyde, washed in 1 mM MgCl2-PBS and incubated in staining solution containing 20× KC (0.82 g K_3_Fe(CN)6 and 1.05 g of K4Fe(CN)6·3H_2_O in 25 mL PBS) 5-Bromo-4-Chloro-3-Indolyl β-D-Galactopyranoside (X-Gal) and 1 mM MgCl_2_ in PBS at pH 5.5 and 37 °C for 3 h. The percentage of positive β-galactosidase cells was quantified using an optical microscope.

### qPCR and qPCR-array

Total RNA was isolated from cells using an RNeasy Kit (Qiagen). 1 μg RNA was retrotranscribed with MultiScribe Reverse Transcriptase (Applied Biosystems). The qPCR reaction was performed using TaqMan Assay (Applied Biosystems) probes for *Actb* (Mm02619580_g1), *Ldha* (Mm01612132_g1), *Bax* (Mm00432051_m1), *Myc* (Mm00487804_m1), *Bmi1* (Mm03053308_g1), *Nanog* (Mm02019550_s1), *p21*^*Cip1*^ (Mm04205640_g1), *Oct3/4* (Mm03053917_g1), *p16*^*INK4a*^ (Mm00494449_m1), *Rest* (Mm00803268_m1), *p15*^*INK4b*^ (Mm00483241_m1), *Sox2* (Mm03053810_s1) *Esrrb* (Mm00442411_m1), *Sox9* (Mm00448840_m1), *Gbx2* (Mm00494578_m1), *Stat3* (Mm0129775_m1), *Hes1* (Mm01342805_m1), *Tbx3* (Mm01195726_m1), *Hey1* (Mm00468865_m1), *Tgfa* (Mm00446232_m1), *Klf2* (Mm00500486_g1), *Tfcp2l1* (Mm00470119_m1), *Klf4* (Mm00516104_m1), *Vegfa* (Mm00437306_m1), *Jarid2* (Mm00445574_m1) and *Ezh2* (Mm00468464_m1). Results are shown as relative quantitative values, which are expressed as 2^-ΔCt^ or as the relative expression of the mRNA.

Applied Biosystems qPCR array cards (ThermoFisher Scientific 4385363) were used to study the differential expression of gene markers for differentiated cells, undifferentiated cells and stem cells and also stemness genes. The quality and quantity of RNA from the cells was measured using a Qubit RNA Assay Kit (ThermoFisher Scientific). Reverse transcription was performed as described before, and cDNA was mixed with Taqman Fast Advanced Master Mix (ThermoFisher Scientific) to conduct qPCR. Z-scores were calculated from the 2^-ΔCt^ data, and the results were relativized to the sample of proliferative MEFs in normoxia. The data are represented as a heatmap using Multi Experiment Viewer (http://www.tm4.org) and as a Venn Diagram using Bioinformatics & Evolutionary Genomics (http://bioinformatics.psb.ugent.be/webtools/Venn/).

### Immunoblotting

Total protein extracts and western blot analysis were performed as described before [18]. The primary and secondary antibodies used were: p16^INK4a^ 1:500 (Santa Cruz; sc-1207), p21^Cip1^ 1:1000 (Abcam; ab109520), p-H2AX (Ser139) 1:1000 (Millipore; 05-636), p-p53 (Ser15) 1:1000 (Abcam; ab1431), Hif1a 1:1000 (BD Transduction Laboratories; 610958), α-tubulin 1:5000 (Sigma-Aldrich; T9026), Sox2 1:1000 (Abcam; ab97959), Nanog 1:500 (Santa Cruz; sc293121), cMyc-tag 1:1000 (Origene; TA150014), Oct3/4 1:500 (Santa Cruz; sc-5279), Tfcp2l1 1:1000 (Novus Biologicals; AF5726), goat anti-rabbit IgG (horseradish peroxidase, HRP) 1:5000 (Abcam; ab97051), rabbit anti-mouse IgG (HRP) 1:5000 (Abcam; ab97046) and rabbit anti-goat IgG (HRP) 1:5000 (Abcam; ab97100). See uncropped WB in supplementary material.

### ChIPmentation and ChIP-qPCR

ChiPmentation, which combines massive DNA sequencing of chromatin immunoprecipitation (ChIP-Seq) samples and the preparation of sequencing libraries with transposase Tn5 (TAGmentation) [19, 20], was performed as described previously [21] with modifications in the crosslinking steps. MEFs from 3T3 experiments in hypoxia and normoxia were used as samples. 2.4 × 10^7^ cells were fixed using a final concentration of 1% paraformaldehyde (PFA) in 200 mM phosphate buffer added to the culture medium and incubated for 10 min at 37 °C. The crosslinking reaction was stopped by adding 125 mM glycine and incubating the cells for 10 min at room temperature. Afterwards, the cells were collected from the culture dish with cold PBS supplemented with protease inhibitors cocktail (PIC) (Merck) and phenylmethylsulfonyl fluoride (PMFS) (Merck). Tfcp2l1 was immuneprecipitated using 2 μg of anti-Tfcp2l1 (Novus Biologicals; AF5726).

The sequencing data was analyzed as described previously [21] and is available through the Gene Expression Omnibus under accession code GSE173648. Additionally, we performed an enrichment analysis for Gene Ontology (GO) terms for Biological Process using Enrichr [22, 23]. We selected for each sample the 20 most significant terms by their p-values and classified them into ancestral ontological terms using the tool CateGorizer [24]. Next, we formed categories defining general biological processes where ancestral terms could be included. The results were represented as the percentage of genes from the number of genes studied.

ChIP-qPCR was used to validate the ChIP-Seq results. The crosslinking steps were done as described above. Once the cells were collected, they were homogenized with lysis buffer (5 mM Pipes, pH 8, 85 mM KCl, 0.5% NP-40, PIC and PMSF). Afterwards, the cells were centrifuged and resuspended in nuclear lysis buffer (1% SDS, 10 mM EDTA, 1.50 mM Tris-HCl pH 8, PIC and PMSF). The resulting chromatin was sonicated in this buffer using a Bioruptor (Diagenode) for 15 min on ice at intervals of 30 s of sonication and 30 s of pause. The size of the DNA fragments was checked by purifying part of the sample. 200 μL of sonicated chromatin was immunoprecipitated in 800 μL immunoprecipitation buffer (0.001% SDS, 1.1% Triton X-100, 1.2 mM EDTA,16.7 mM Tris-HCl pH 8.1,167 mM NaCl, PIC and PMSF) with antibodies against Tfcp2l1 (Novus Biologicals; AF5726) or RNA polymerase II (POLR2A) (ThermoFisher Scientific; MA1-10882), which was used as a positive control. After 16 hours of incubation, ChIP-Grade Protein G Magnetic Beads (Cell Signaling) were added to the mixture, which was washed with low salt washing buffer (0.1% SDS, 1% Triton x-100, 2 mM EDTA, 20 mM Tris-HCl, pH 8.1, and 150 mM NaCl), high salt washing buffer (0.1% SDS, 1% Triton x-100, 2 mM EDTA, 20 mM Tris-HCl, pH 8.1, and 500 mM NaCl) and LiCl washing buffer (0.25 M LiCl, 1% NP-40, 1% NaDoc, 1% EDTA and 10 mM Tris-HCl, pH 8.1) using a magnetic rack. Magnetic beads were removed by washing with TE and 1% TE-SDS buffer and heating the mixture at 65 °C.

Finally, the DNA was purified using a standard ethanol precipitation protocol. The DNA was amplified by qPCR using primers against sequences where the ChIP-seq peaks were located. A gene desert locus was used as a negative control, and the first exon of α-tubulin was used as a positive control for the qPCR. Ct data was analyzed using the relative quantification method, and the results (expressing the proportion of DNA of the sample to the input) was represented by normalizing the sample immunoprecipitated with anti-Tfcp2l1 to the sample immunoprecipitated with IgG. The primers used were *Sox2* (Fw: ACCAGGGCTGTGTTAAATGC Rv: GAGTGAGCAGGGGAAGGAAC), *Sox9* (Fw: TTTTGGAGCAGGGAGGGTTG, Rv: gtgtgtgtgtgtTGAGAGGG), *Tgfa* (Fw: CATCCACTGGTGCTGCTAAG, Rv: GGTCCTCAGTGTAGTCCAGG), *Gene desert* (Fw: CACTCTTCTTATAGGACCCTTTG Rv: CTGTCTACCTGTTCTTTACATTCT), and *α-Tubulin* (Fw: GCACCAATCACCACTCTCCT, Rv: AGGCGGTCGATGTAAGAGAA).

### Analysis of public datasets

To analyze the regulator regions associated with *Tfcp2l1* in the DNA, the chromatin 3D structure was observed using HiC data (ENCODE, GSE74055) [25] in the Wash U Epigenome Browser. Potential regulator elements (active promoters and enhancers) were analyzed using public ChIP-Seq data for histone modifications, H3K4me3 and H3K27ac (GSE49847) [26]. These datasets were visualized in USCS Genome Browser, and peaks included in the region studied using the HiC data were analyzed with JASPAR [27] to determine if Hif1α, Arnt or the Hif1α-Arnt DNA binding domains were contained in the sequence. Only peaks where predictions to find these DNA-binding motifs resulted in score values higher than 8 were selected.

Chipmentation data generated in this study is available through the Gene Expression Omnibus under accession code GSE173648.

Retrospective meta-analysis of transcriptome profiling of iPSCs reprogrammed under different oxygen concentrations (GSE26489) and their reprogramming intermediates (GSE116309), microarray experiments were previously performed in Shinya Yamanaka laboratory using SurePrint G3 Mouse Gene Expression 8×60 Kit (Agilent Technology) in a Microarray Scanner System (Agilent Technology). Results were obtained analyzing this data using GeneSpring (Agilent Technology). Additional data are available from Dr. Shinya Yamanaka or Dr. Yoshinori Yoshida upon reasonable request.

### ATAC-seq

ATAC-seq assays were performed using standard protocols [20, 28], with minor modifications. Briefly, 70,000 MEFs per condition were collected by centrifugation for 5 min at 500 × *g* 4 °C. Supernatant was removed and cells washed with PBS. Then, cells were lysed in 50 µl of Lysis Buffer (10 mM Tris-HCl pH 7.4, 10 mM NaCl, 3 mM MgCl_2_, 0.1% NP-40, 1x Roche Complete protease inhibitors cocktail) by pipetting up and down. The whole cell lysate was used for TAGmentation, which were centrifuged for 10 min at 500 × *g* 4 °C and resuspended in 50 µl of the Transposition Reaction, containing 2.5 µl of Tn5 enzyme and TAGmentation Buffer (10 mM Tris-HCl pH 8.0, 5 mM MgCl_2_, 10% w/v dimethylformamide), and incubated for 30 min at 37 °C. Immediately after TAGmentation, DNA was purified using the Minelute PCR Purification Kit (Qiagen) and eluted in 20 µl. Libraries were generated by PCR amplification using NEBNext High-Fidelity 2X PCR Master Mix (NEB). The resulting libraries were multiplexed and sequenced in a HiSeq 4000 pair-end lane producing 100 M of 49-bp pair end reads per sample.

ATAC-seq reads were aligned to the GRCm38 (mm10) mouse genome assembly using Bowtie2 2.3.5 [29] and those pairs separated by more than 2 Kb were removed. The Tn5 cutting site was determined as the position −4 (minus strand) or +5 (plus strand) from each read start, and this position was extended 5 bp in both directions. Reads below 150 bp were considered nucleosome free. Conversion of SAM alignment files to BAM was performed using Samtools 1.9 [30]. Conversion of BAM to BED files, and peak analyses, such as overlaps or merges, were carried out using the Bedtools 2.29.2 suite [31]. Conversion of BED to BigWig files was performed using the genomecov tool from Bedtools and the wigToBigWig utility from UCSC [32]. For visualization purposes, reads were extended 100 bp. Peaks were called using MACS2 2.1.1.20160309 algorithm [33] with an FDR < 0.05, and merged into a single pool of peaks that was used to calculate the differentially accessible sites with the DESeq2 1.18.1 package in R 3.4.3 [34], using pseudo-replicates by randomly splitting reads from each experiment in two files. For data comparison, all ATAC-seq experiments used were normalized using reads falling into peaks to counteract differences in background levels between experiments [21]. Heatmaps, average profiles and k-means clustering of ATAC-seq data were generated using computeMatrix and plotHeatmap tools from the Deeptools 3.5 toolkit [35]. TF motif enrichment were calculated using Homer [Heinz, Mol Cell 2010], with standard parameters. For gene assignment to ATAC peaks, we used the GREAT 3.0.0 tool [36], with the basal plus extension association rule with standard parameters (5 Kb upstream, 1 Kb downstream, 1 Mb maximum extension).

### Statistical information

Experiments were performed a minimum of three independent times (biological replicates) with three technical replicates each time. The values of the results plotted represent the mean and error bars, which correspond to standard error of mean (SEM). Student’s t Test and ANOVA (one way) analysis were performed using GraphPad Prism 6.01. Statistical significance is represented as *p < 0.05, **p < 0.01 and ***p < 0.001.

## Results

### Hypoxia bypasses cellular senescence

**Fig. 1:**
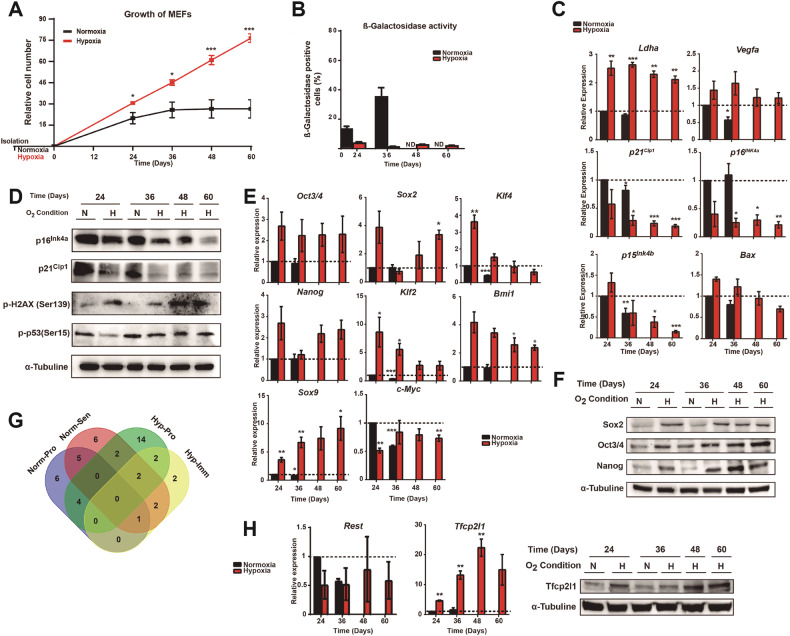
Hypoxia can extend lifespan in vitro and increase the expression of stemness-associated genes. **A** Growth of MEFs in normoxia or hypoxia. MEFs were isolated and cultured in hypoxia or nomoxia for their amplification. Growth was observed using the 3T3 protocol. **B** Percentage of acid β-galactosidase activity positive cells. MEFs were stained with X-Gal. **C** Transcriptional analysis of the expression of genes related to hypoxia or cellular senescence. The mRNA levels of *Ldha, Vegfa, p21*^*Cip1*^*, p16*^*INK4a*^*, p15*^*INK4b*^ and *Bax* were measured by qRT-PCR. **D** Protein levels of senescence markers. Protein levels of p16^INK4a^, p21^Cip1^, p-H2AX (Ser139) and p-p53 (Ser15) were compared by western blot using α-tubulin as the load control. **E** Expression of stemness-associated genes. mRNA levels of *Oct3/4, Sox2, Klf4, Nanog, Klf2, Bmi1, Sox9 and cMyc* were measured by qPCR 24, 36, 48 and 60 days after the starting 3T3 modified protocol. **F** Levels of stemness-associated proteins. Protein levels of Sox2, Oct3/4 and Nanog were compared by western blot using α-tubulin as the load control. **G** Classification of the number of genes overexpressed by each sample. The Venn diagram shows the number of genes overexpressed by each sample. **H** Validation of *Rest* and *Tfcp2l1* overexpression. The mRNA levels of both genes were measured by qPCR. Protein levels were also measured for Tfcp2l1 by western blot. Graphs show the average of at least, 3 biological and experimental independent repetitions. Student’s t test was used for the growth and mRNA analysis and ANOVA (one way) analysis was used for the acid β-galactosidase analysis. Graphs show the average of at least, 3 biological and experimental independent repetitions (*p < 0.05; **p < 0.01; ***p < 0.001). ND not determined.

To study the effects of hypoxia on cellular senescence, we compared the proliferation and senescence features of MEFs in two different oxygen conditions: normoxia (20% O_2_) and hypoxia (3% O_2_). First, we isolated and cultured MEFs from day 13.5 embryos in either condition following a modified 3T3 protocol. MEFs extracted in normoxia entered senescence around day 30, but in hypoxia they remained immortal (Fig. 1A). Furthermore, the senescent MEFs showed higher acid β-galactosidase activity and acquired typical senescent morphological features, whereas those cultured in hypoxia did not (Fig. 1B and Supplementary Fig. S1).

Next, we measured the expression levels of different senescent markers. Two transcriptional targets of Hifα proteins, *Ldha* and *Vegfa* [37], showed increased expression levels in hypoxia (Fig. 1C). On the other hand, the expression of several senescent markers, including the cyclin-dependent kinase inhibitors (CKIs) *p16*^*INK4a*^, *p15*^*INK4b*^ and *p21* ^*CIP1*^, was higher in normoxia, and their expression decreased as the cells continued proliferating (Fig. 1C, D). We also found high levels of phosphorylated H2AX (Ser 139) and phosphorylated p53 (Ser 15) in hypoxia, which could indicate DNA damage produced by excessive proliferation [38–40]. However, we also found a decrease in the expression of p53 targets, such as the proapoptotic protein *Bax* and *p21*^*Cip1*^ [41] (Fig. 1C, D) suggesting that the transcriptional response to p53 is impaired in MEFs in hypoxia. Altogether, these data indicate that MEFs bypass senescence in hypoxic conditions.

### Hypoxia induces stemness in naïve MEFs

Hypoxia increases the lifespan of differentiated fibroblasts, mimicking the unlimited replicative potential of stem cells [10] (Fig. 1A). Therefore, we investigated whether hypoxia promotes the stem cell properties of MEFs. We found increased expression levels of three Yamanaka factors (*Oct3/4*, *Sox2*, and *Klf4*) along with *Nanog*, *Klf2, Bmi1* and *Sox9* (Fig. 1E). We also found that the mRNA level of *cMyc* was maintained in hypoxia but decreased with time in normoxia (Fig. 1E). We also found a correlation in mRNA and protein levels for Sox2, Oct3/4 and Nanog (Fig. 1F). These results confirmed the acquisition of a stem cell expression profile in hypoxia and could explain the increase in the proliferative capacity of the MEFs.

To explore which other stemness genes are involved in the immortalization phenotype caused by hypoxia, we used a qPCR array that included genes that are markers for differentiated cells, undifferentiated cells, stem cells and also stemness genes. We compared the expression profile of MEFs at two different time points: when cells showed similar replicative properties in normoxia and hypoxia (Norm-Pro and Hyp-Pro); and when the cells entered senescence in normoxia but still proliferated and were considered immortalized in hypoxia (Norm-Sen and Hyp-Imm). Each of the four conditions showed a unique expression profile in the qPCR array (Supplmentary Fig. S2). Hyp-Pro and Hyp-Imm cells generally overexpressed more genes related to undifferentiated cells and stem cells compared to Norm-Pro and Norm-Sen, which generally expressed more gene markers for differentiated cells. Next, we focused on the genes highly expressed in Hyp-Imm cells, which could regulate immortalization. Two genes were overexpressed, and both were related to stem cells: *Rest* and *Tfcp2l1* (Supplmentary Fig. S2). *Tfcp2l1* was increasingly expressed with time in hypoxic MEFs at the mRNA and protein levels (Fig. 1G, H).

### *Tfcp2l1* in MEF immortalization

**Fig. 2:**
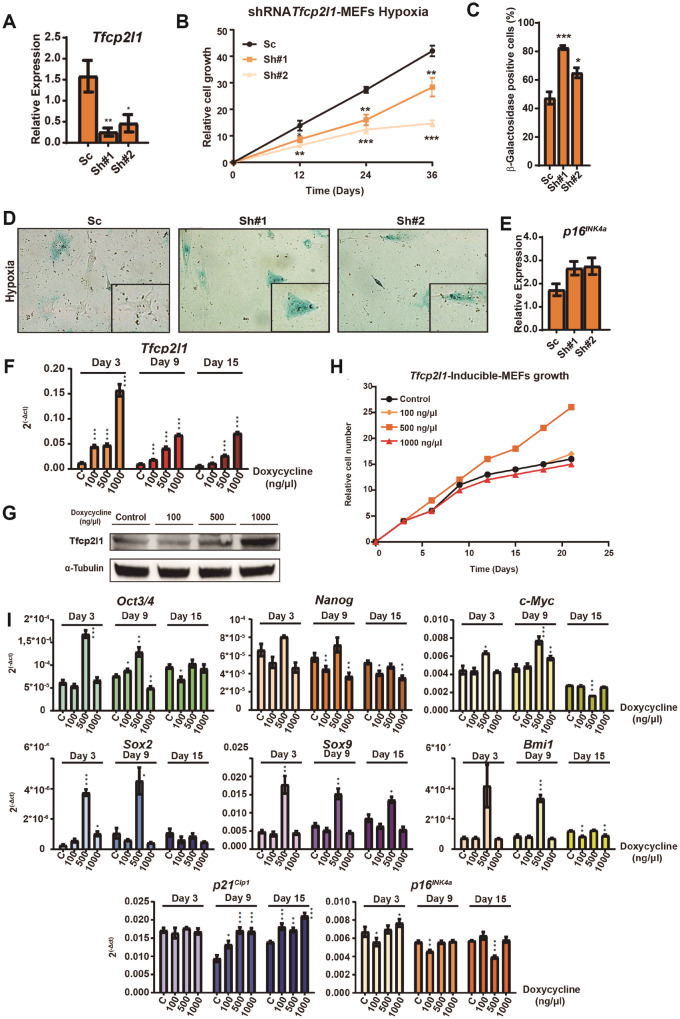
Reduction in the expression of *Tfcp2l1* induces premature senescence with high levels of *p16*^*INK4a*^. **A** mRNA levels of Tfcp2l1 in shRNA-MEFs. MEFs were cultured in hypoxia and transduced with a scrambled shRNA or shRNA against *Tfcp2l1* mRNA. 3T3 protocol started after selection and transcriptional expression of *Tfcp2l1* was measured by qRT-PCR at day 3. **B** Relative cell growth of MEFs-Sc, MEFs-Sh#1 and MEFs-Sh#2. After selection, relative cell growth was observed using a 3T3 modified protocol. **C** β-galactosidase activity in hypoxia. Transduced and selected MEFs were fixed after 3 days of culture in the 3T3 modified protocol and stained with X-Gal. **D** Senescent-associated morphology and X-Gal staining. Cellular morphology was observed while quantifying acid β-galactosidase activity. **E** The transcriptional expression *p16*^*INK4a*^ was measured by qRT-PCR at day 3 of 3T3 protocol. **F** mRNA levels of *Tfcp2l1*. MEFs were cultured in normoxia and transduced with *Tfcp2l1* cDNA and a vector expressing a transactivator inducible by doxycycline. After the selection of double transduced cells, MEFs were cultured in a 3T3 modified protocol. mRNA levels of *Tfcp2l1* were analyzed by qPCR on days 3, 9 and 15 of the protocol. The representative experiment shown was performed in independent triplicate samples. **G** Protein levels of *Tfcp2l1* in MEFs. MEFs were treated with different concentrations of doxycycline during the 3T3 modified protocol, and protein levels of Tfcp2l1 were measured by western blot. **H** Growth of MEFs-. Transduced and selected MEFs-*Tfcp2l1*-inducible treated with doxycycline in normoxia were cultured in the 3T3 modified protocol. Relative cell numbers are shown. **I** mRNA levels of stemness-associated genes and CKIs. mRNA levels of *Oct3/4, Nanog, cMyc, Sox2, Sox9, Bmi1, p21*^*Cip1*^ and *p16*^*INK4a*^ were analyzed by qPCR. Student’s t test was used for the statistical analysis of relative cell growth and mRNA levels in MEFs. ANOVA (one way) was used for the statistical analysis of acid β-galactosidase activity. The representative experiment shown was performed in independent triplicate samples. Graphs show the average of at least 3 biological and experimental independent repetitions (*p < 0.05; **p < 0.01; ***p < 0.001).

Because *Tfcp2l1* plays an important role in maintaining the self-renewal and pluripotency of stem cells [42, 43], we decided to study its causality and relevancy in the lifespan extension of MEFs in hypoxia. To do this, we modified its expression in hypoxia. First, we induced the overexpression of two different shRNAs against the mRNA of *Tfcp2l1* in MEFs cultured in hypoxia and normoxia, as control (Fig. 2A). Physiologically, we observed a sudden stop of proliferation in the MEFs overexpressing the shRNAs, whereas MEFs expressing a scrambled shRNA showed unchanged growth (Fig. 2B). We also observed increased acid β-galactosidase activity along with the acquisition of typical senescent morphology in MEFs with low levels of *Tfcp2l1* (Fig. 2C, D). This pattern was consistent with the downregulation of *Tfcp2l1* in normoxic MEFs (Supplementary Figs. S3B–4D). The downregulation of *Tfcp2l1* was accompanied by an increased expression of *p16*^*INK4a*^ in both oxygen conditions (Fig. 2E), confirming cell cycle arrest and senescence. These findings suggest that *Tfcp2l1* is necessary for maintaining the proliferation capacity of MEFs, and its downregulation activates senescence.

To determine if the ectopic expression of *Tfcp2l1* in normoxia could replicate the lifespan increase in MEFs observed in hypoxia, we induced the constitutive expression of *Tfcp2l1* cDNA in normoxia. MEFs highly overexpressing *Tfcp2l1* (*Tfcp2l1*-MEFs) stopped proliferating, acquiring typical morphological features of senescent cells and also increased their acid β-galactosidase activity (Supplmentary Fig. S3A, B). We confirmed that *Tfcp2l1* was overexpressed at the mRNA and protein levels. We noticed that at mRNA level, however, the overexpression of *Tfcp2l1* was considerably higher (1000 times) than in hypoxia. This could explain the proliferation arrest, as we also found higher expressions of *p16*^*INK4a*^ and *p21*^*Cip1*^ in *Tfcp2l1*-MEFs (Supplmentary Fig. S3C, D). *Oct3/4*, *Sox2*, *Klf4* and *Nanog* all had higher expression levels. We also observed the upregulation of genes related to Tfcp2l1 signaling in mouse (m)ESCs and to development, including *Esrrb*, *Klf2*, *Tbx3* and the Notch-related genes *Hes1* and *Hey1* [42–44] (Supplmentary Fig. S3D). These results suggest that supraphysiological levels of *Tfcp2l1* are an oncogenic stimulus that induce an oncogene-induced senescence (OIS)-like response in normal cells.

Next, we tried to reproduce in normoxia the physiological upregulation of *Tfcp2l1* seen in hypoxia using a doxycycline-inducible expression system. We confirmed that the overexpression of *Tfcp2l1* was proportional to the concentration of doxycycline added to the culture medium (Fig. 2F, G). Of the three doxycycline doses tested, only 500 ng/μL increased Tfcp2l1 protein at physiological range levels and increased the MEF lifespan (Fig. 2H, and supplementary Fig. S4). This dose in normoxia also upregulated *Oct3/4*, *cMyc*, *Nanog*, *Sox2*, *Sox9* and *Bmi1* at days 3 and 9 (Fig. 2I). Finally, *p21*^*Cip1*^ expression increased, but *p16*^*INK4a*^ expression was unchanged (Fig. 2I). These results indicate that only physiological or near physiological expression levels of *Tfcp2l1* extend the lifespan of MEFs in normoxia by regulating stem cell genes and CKIs.

### Activation of *Tfcp2l1* in hypoxia

**Fig. 3:**
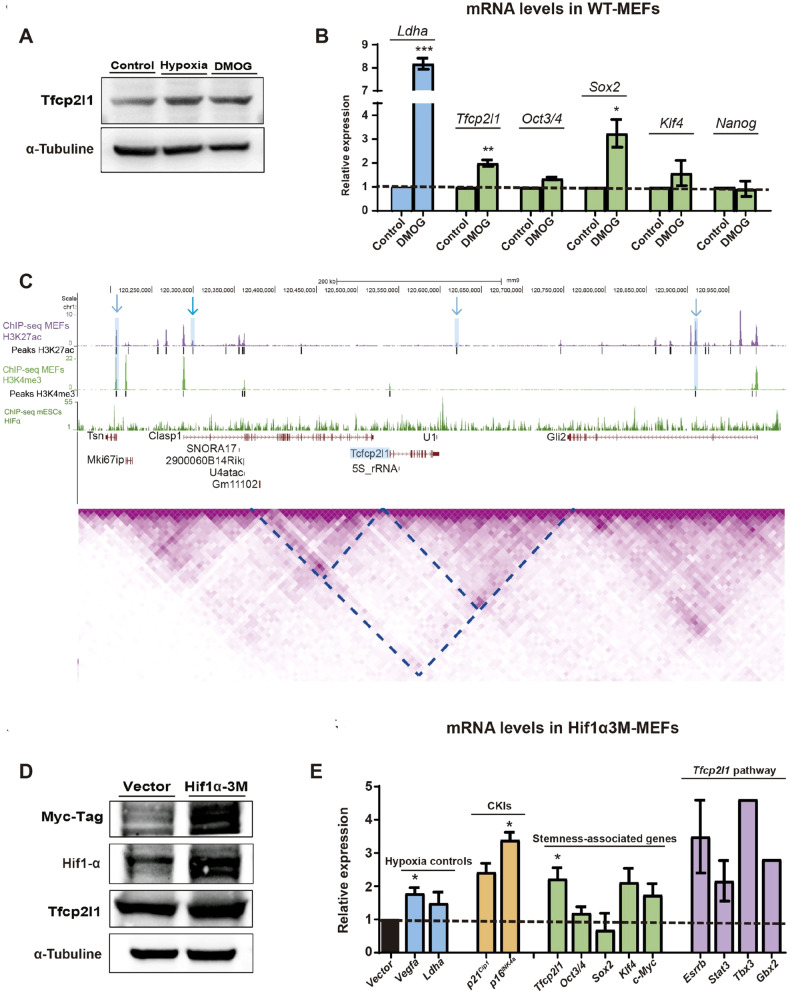
Activation of Hifα in normoxia promotes the expression of *Tfcp2l1*. **A** Tfcp2l1 protein levels in MEFs treated with DMOG in normoxia. MEFs were treated with DMOG for 24 h in normoxia, and proteins were extracted and compared by western blot with control MEFs in normoxia and hypoxia. **B** mRNA levels of MEFs treated with DMOG. MEFs were treated with DMOG for 24 h in normoxia, and mRNA was extracted and compared by qRT-PCR with control MEFs in normoxia. **C** Potential promoters and enhancers at the Hif1α DNA-binding motif. HiC data was visualized to locate TADs near *Tfcp2l1*. DNA sequences from peaks of H3K27ac and H3K4me3 ChiP-Seq in MEFs were analyzed with JASPAR to detect the Hif1α, ARNT and the ARNT-Hif1a DNA binding domains. Arrows show peaks with higher probability to find these DNA binding motifs. **D** Protein levels of Tfcp2l1 and Hif1α induced after Hi1α3M transduction. Protein levels of Hif1α, Myc-Tag, and Tfcp2l1 were measured by western blot after the transduction. **E** mRNA levels of hypoxia target genes, CKIs, stemness-associated genes and genes from the Tfcp2l1 pathway in development. Transcriptional expression was measured by qRT-PCR for *Vegfa, Ldha, p16*^*INK4a*^*, p21*^*Cip1*^*, Tfcp2l1, Oct3/4, Sox2, Klf4, cMyc, Esrrb, Stat3, Tbx3* and *Gbx2*. Student’s t test was used for the statistical analysis of relative cell growth and mRNA levels in MEFs. Graphs show the average of at least 3 biological and experimental independent repetitions (*p < 0.05; **p < 0.01; ***p < 0.001).

Next, we investigated how hypoxia activates *Tfcp2l1*. First, we simulated hypoxia by adding to the culture medium of normoxic MEFs dimethyloxalylglycine (DMOG), a HIF-hydroxilase inhibitor which allows the stabilization of HIFα proteins in normoxia. We observed the overexpression of *Tfcp2l1* in these hypoxia-like conditions (Fig. 3A). DMOG also stimulated a higher expression level of *Ldha*, indicating Hifα protein activity (Fig. 3B). We also observed an increased expression of *Oct3/4*, *Sox2* and *Klf4* (Fig. 3B). Therefore, we investigated Hif1α DNA binding motifs in cis-regulatory elements close to *Tfcp2l*1. For this purpose, we used HiC public data [25]. We delimited the DNA region of study to the topologically associated domains (TADs) surrounding *Tfcp2l1*. We noticed that *Tfcp2l1* shares a potential TAD with another important gene for development, *Gli2*. However, this TAD is divided into two sub-TADs. Then, we studied potential active enhancers and promoters in the area covered by these sub-TADs using ChIP-Seq public data for H3K27ac and H3K4me3, which mark active enhancers and promoters or only promoters, respectively [26, 45]. We then used JASPAR to find the DNA-binding motif of Hif1α in the sequences of the peaks. We found four potential active enhancers and promoters in the studied area containing that motif with a high score, one of them downstream of *Tfcp2l1*, that would likely be regulating its expression through Hif1α binding. Indeed, one of them shows Hif1α binding in mouse embryonic stem cells (mESCs) and Hif1α is also bound close to the *Tfcp2l1* gene in these cells (Fig. 3C).

To confirm if Hif1α activated the expression of *Tfcp2l1*, we overexpressed a mutant version that is stable and constitutively active in normoxia (Hif1α-3M) [46]. As result, we observed increased expression of *Vegfa* and *Ldha*, which confirmed the transcriptional activity of Hif1α in normoxia. We observed an increase in the mRNA levels of *p16*^*INK4a*^ and *p21*^*Cip1*^ when MEFs stopped growing with features of OIS after overexpressing Hif1α-3M. Additionally, *Tfcp2l1* overexpression was detected at the mRNA and protein levels, and we observed a higher expression of some stemness genes and genes implicated in the Tfcp2l1 signaling pathway in development (Fig. 3D–E). All these data strongly suggest that Hif1α regulates the expression of *Tfcp2l1* under hypoxic conditions.

### *Tfcp2l1* in cellular reprogramming

**Fig. 4:**
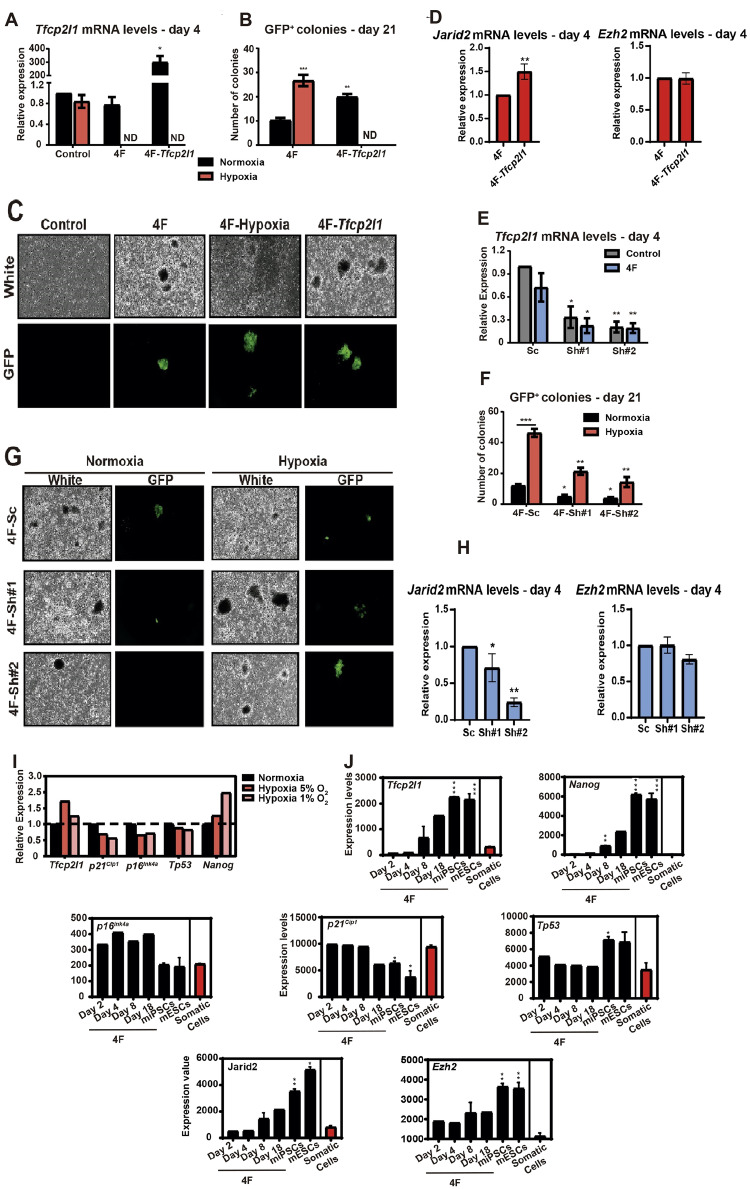
*Tfcp2l1* can replicate the effect of hypoxia in reprogramming. **A** Transcriptional expression of Tfcp2l1. 4 days after transduction, mRNA samples were obtained from all conditions, and qRT-PCR was performed. **B** Number of GFP^+^ iPSCs colonies. GFP^+^ colonies from MEFs were counted 21 days after the transduction in all conditions. **C** iPSCs colonies. GFP^+^ colonies from MEFs were observed. Fluorescent microscopy images are shown. **D** Transcriptional expression of *Jarid2* and *Ezh2*. 4 days after transduction, mRNA samples were obtained and qRT-PCR of Tfcp2l1 transcriptional targets was performed. **E** Transcriptional expression of *Tfcp2l1* in MEFs expressing *Tfcp2l1* shRNA. 4 days after the transduction, mRNA samples were obtained from all conditions, and qRT-PCR was performed. **F** Number of GFP^+^ iPSCs colonies in MEFs expressing *Tfcp2l1* shRNA. GFP^+^ colonies from MEFs were counted 21 days after the transduction in all conditions. **G** iPSC colonies. GFP^+^ colonies from MEFs were observed. Fluorescent microscopy images are shown. **H** Transcriptional expression of *Jarid2* and *Ezh2*. 4 days after transduction, mRNA samples were obtained from 4F-Sc and 4F-shRNAs-MEFs and qRT-PCR of Tfcp2l1 transcriptional targets was performed. **I** Transcriptional expression of iPSCs. Data from experiments performed in Yamanaka-lab was analyzed and mRNA levels of *Tfcp2l1*, *p16*^*INK4a*^, *p21*^*Cip1*^, *Tp53* and *Nanog* in iPSCs cultured in hypoxia (5% O_2_ and 1% O_2_) and normoxia was compared. **J** Transcriptional expression during the reprogramming process and in mESCs. Data from experiments performed in Yamanaka-lab was analyzed and mRNA levels of *Tfcp2l1, Nanog*, *p16*^*INK4a*^, *p21*^*Cip1*^, *Tp53*, *Jarid2* and *Ezh2* during reprogramming process (days 2, 4, 8 and 18) in normoxia was compared to mRNA levels in mESCs and somatic cells. The number of biological replicates for experiments 4I and 4J is 2, each one in independent triplicate samples. Student’s T test was used for mRNA analysis. Graphs show the average of, at least, 3 biological and experimental independent repetitions (*p < 0.05; **p < 0.01; ***p < 0.001).

Considering the role of *Tfcp2l1* in the regulation of proliferation and self-renewal of ESCs and the results obtained in our experiments, we decided to study the relevance of *Tfcp2l1* in the increase of cellular reprogramming caused by hypoxia [17]. To do this, we transduced the 4 Yamanaka factors, *Oct3/4*, *Sox2*, *Klf4* and *cMyc* (4F), and 4F plus *Tfcp2l1* in MEFs. We confirmed the overexpression of *Tfcp2l1* 4 days after the transduction (Fig. 4A). We then compared the number of iPSC colonies generated in 4F-MEFs and 4F-Tfcp2l1-MEFs cultured in normoxia with 4F-MEFs cultured in hypoxia for 9 days. All MEFs for these experiments carried a GFP reporter cassette for the expression of *Nanog* [47]. We observed an increase in GFP^+^ colonies in 4F-Tfcp2l1-MEFs, similar to the increase in 4F-MEFs treated with hypoxia (Fig. 4B, C). We also checked the expression of Tfcp2l1 transcriptional targets in these conditions and found higher levels of *Jarid2* but not of *Ezh2* in 4F-Tfcp2l1 MEFs (Fig. 4D).

Additionally, we performed reprogramming experiments using the two shRNAs against *Tfcp2l1* mRNA in MEFs described above. We confirmed reduced *Tfcp2l1* mRNA levels in MEFs (Fig. 4E) and then compared the number of iPSCs generated in hypoxia and normoxia 21 days after the transduction. We observed that in normoxia and hypoxia, both shRNAs decreased the number of GFP^+^ colonies compared to 4F-MEFs transfected with scrambled shRNA (Fig. 4F, G). Importantly, we noticed that the expression of shRNA in 4F-MEFs treated with hypoxia reduced the number of GFP^+^ colonies to a similar number as GFP^+^ colonies obtained from 4F-MEFs in normoxia. Here, the ectopic expression of both shRNAs against *Tfcp2l1* reduced the mRNA levels of *Jarid2* and produced a slight decrease in *Ezh2* in the case of shRNA #2 (Fig. 4H).

Finally, using transcriptional data from the reprogramming experiments, we investigated the expression of different genes modulated by hypoxia. We observed that culture at 5% O_2_ or 1% O_2_ increased the expression of *Tfcp2l1* and *Nanog* but reduced the expression of *p16*^*INK4*^*a* and *p21*^*Cip1*^ and also *Tp53* (Fig. 4I). Additionally, we investigated the expression of these genes during the reprogramming process in normaxia and compared their mRNA levels at different days in miPSCs and mESCs and somatic cells. Again, the expression of *Tfcp2l1* was increased in miPSCs during the whole reprogramming process and in mESCs compared to somatic cells. The same expression pattern was observed for *Nanog*. Like MEFs cultured in hypoxia, the expression of *p21*^*Cip1*^ and *p16*^*INK4a*^ was reduced during the reprogramming process, while *Tp53* was increased. Additionally, *Jarid2* and *Ezh2* showed the same expression pattern as *Tfcp2l1* (Fig. 4J). Together, our findings suggest that *Tfcp2l1* plays a relevant role in the reprogramming observed in hypoxia, possibly by regulating pluripotency and proliferation.

### Transcriptional targets of Tfcp2l1 during immortalization by hypoxia

**Fig. 5:**
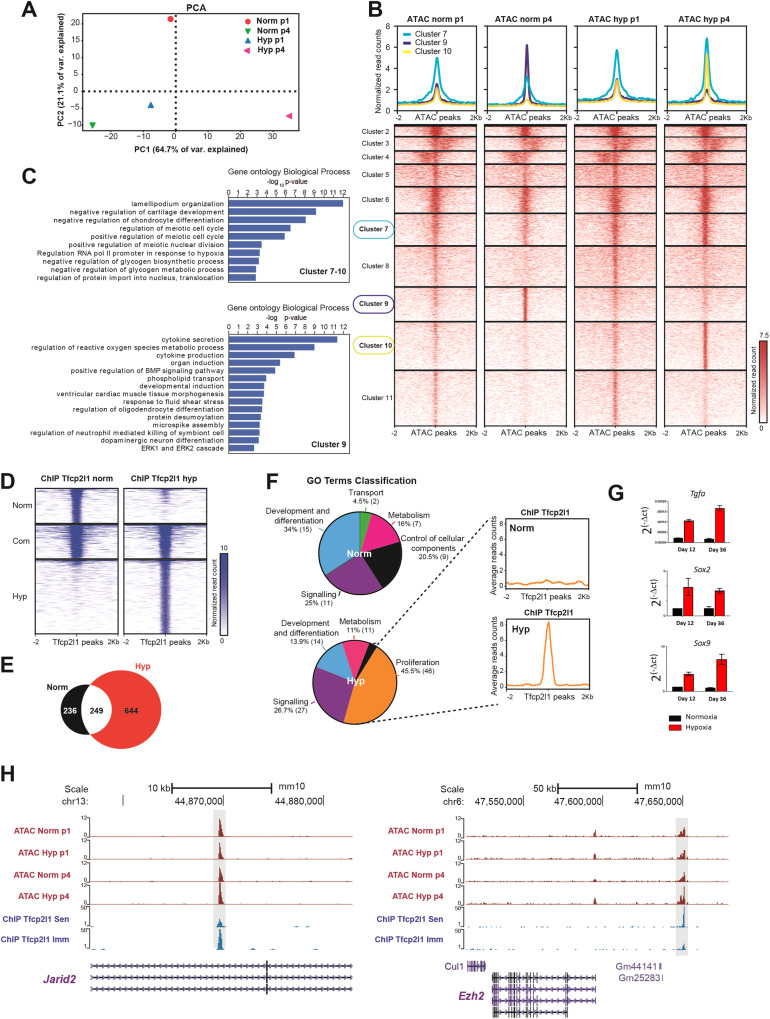
Tfcp2l1 regulates proliferation and stemness genes at the chromatin level. **A** PCA analyses of ATAC-seq samples. ATAC-seq experiments in proliferating MEFs both in normoxia and hypoxia, as well as senescent and immortalized MEFs are shown. **B** Clustering of open chromatin regions with Tfcp2l1 motifs. ATAC-seq peaks containing Tfcp2l1 motifs were clustered with the *k*-means method to find condition-specific peaks. **C** Gene Ontology analyses. Genes associated with ATAC peaks of clusters 9 and 7–10 were subjected to GO enrichment analyses of biological processes. Terms with an enrichment FDR < 0.05 are shown. **D** ChIP-seq of Tfcp2l1 in senescent vs. immortalized MEFs. Heatmaps showing normalized reads for ChIP-seq peaks common or specific to senescent and immortalized MEFs. **E** Venn diagram for genes associated to the peaks of each sample. The number of genes specifically associated to the peaks of each sample are shown, and the intersection of the graph shows the number of genes commonly associated to both samples. **F** Enrichment of proliferation genes associated with Tfcp2l1 binding in immortalized MEFs. *Left*, classification of genes included in the most represented Biological Process GO Terms in general functional categories. After GO Term enrichment analysis for Biological Process, the 20 most significantly enriched terms were classified using CateGOrizer into ancestral terms. These ancestral terms were also classified in more general biological processes. The number of genes in each category and percentage from total genes found in the GO Terms analysis are shown. *Right*, average sequencing signal for peaks included in the Proliferation functional category. The average read counts for the 46 genes included in the functional category Proliferation is represented in the graph comparing Norm and Hyp. **G** mRNA levels of *Jarid2* and *Ezh2* in MEFs. mRNA was obtained from MEFs in 3T3 experiments in hypoxia and normoxia on days 24 and 36, and qPCR was performed. Ct data were analyzed using the relative quantification method. **H** ATAC-seq and ChIP-seq genomic tracks. ATAC-seq and ChIP-seq signals in the analyzed conditions are shown for the *Jarid2* and *Ezh2* gene loci.

At this point, we explored transcriptional targets of Tfcp2l1 that could be activated in hypoxia promoting the immortalization of MEFs. For this, we first performed chromatin accessibility analyses by the Assay for Transposase-Accessible Chromatin with high throughput sequencing (ATAC-seq) in proliferating MEFs both in normoxia and hypoxia, as well as senescent and immortalized MEFs. Principal component analyses of the samples showed differences among samples that were higher between senescent and immortalized MEFs (Fig. 5A). We called peaks to identify putative cis-regulatory elements (CREs) and performed differential accessibility analyses. This led to the identification of 1945 and 2826 open chromatin regions with increased or decreased accessibility, respectively, in immortalized vs. senescent MEFs (Supplementary Fig. S5A, B). Motif enrichment analyses identified the Tfcp2l1 motif significantly enriched only in the regions with increased accessibility in immortalized MEFs (Supplementary Fig. S5C), suggesting a role of Tfcp2l1 in chromatin opening in hypoxia. Therefore, we decided to focus on ATAC peaks containing Tfcp2l1 motifs, which were 19,246 out of 166,293 total peaks (11.6%). Clustering of these peaks with the *k*-means method identified 11 clusters, three of which being particularly interesting ones: cluster 9, containing peaks with more accessible chromatin in senescent MEFs, and clusters 7 and 10, containing peaks with more accessible chromatin in immortalized MEFs (Fig. 5B). We assigned these CREs with their putative target genes and analyzed the enrichment of Biological Process Gene Ontology (GO) terms. We found that genes associated to cluster 9 peaks were enriched in cytokine secretion, among other functions, which is consistent with a senescence-associated secretory phenotype (Fig. 5C). On the other hand, genes associated to peaks of clusters 7 and 10 were enriched in differentiation and metabolic processes, as well as in the regulation of transcription in response to hypoxia (Fig. 5C). These data suggest a role of Tfcp2l1 in the regulation of senescence and immortalization that is consistent with our observations.

Next, we performed chromatin immunoprecipitation with high-throughput sequencing (ChIP-Seq) in senescent MEFs from normoxia (Norm) and MEFs immortalized by hypoxia (Hyp). The ChIP-Seq data revealed Tfcp2l1 peaks associated to 249 genes common to both normoxic and hypoxic MEFs, 236 genes specific to Norm, and 644 specifics to Hyp (Fig. 5D, E). These data indicate that hypoxia leads to a redistribution of *Tfcp2l1* binding compared to senescence. Additionally, we found an enrichment of the DNA-binding motif of *Tfcp2l1* in the common peaks and in the Hyp peaks, validating the specificity of the peaks obtained (Supplementary Fig. S6A). Next, we searched for functional enrichments in the genes associated to the peaks in each sample. First, we studied GO Terms for Biological Process, categorizing the 20 most significative terms found in general biological functions. For the normoxia senescent sample (236 + 249 genes), we only found few genes annotated to the enriched terms. The most enriched category referred to development and differentiation processes, but contained only 15 genes. However, for hypoxic MEFs (644 + 249 genes), we found 46 genes with functions in the regulation of proliferation (Fig. 5F). The peaks found in putative regulatory regions associated to these 46 genes showed a very high and specific signal compared to normoxic MEFs (Fig. 5F). We validated by ChIP-qPCR the binding of Tfcp2l1 to the sequences where we found the peaks of these genes in MEFs immortalized by hypoxia (Supplementary Fig. S5B). Here, we found common peaks associated to the Polycomb components *Jarid2* and *Ezh2, Tgfa*, a marker of hypoxia, as well as stemness genes as *Sox2* and *Sox9* (Fig. 5G, H and Supplementary Fig. S7). As shown previously, *Sox2* and *Sox9* were overexpressed in hypoxia compared to normoxia at different points of the lifespan of MEFs (Fig. 1E). We show now (Fig. 5G) the transcriptional activation of *Tgfa, Sox2 and Sox9* at the same hypoxic conditions as the pics observed in Chip-seq. And, we confirmed that the mRNA expression of the Polycomb components *Jarid2*, and *Ezh2*, were also transcriptionally induced in hypoxia (Supplementary Fig. S8).

Altogether, these data show that Tfcp2l1 has a role in the immortalization of MEFs by hypoxia by transcriptionally controlling genes related to the response to hypoxia, proliferation and stemness. During IP generation in normoxia, Jarid2 increases transcription by expression of Tfcp2l1, at difference of Ezh2 (Fig. 4D). Furthermore, downregulation of Tfcp2l1 by specific siRNA, also downregulated Jarid2 but not Ezh2 (Fig. 4H). Therefore, we focus on Jarid 2 as possible mediator of Tfcp2l1 during immortalization.

To finally confirm the functional significance of *Jarid2*, we tested whether *Jarid2* acts as a critical mediator of hypoxia/*Tfcp2l1* facilitates immortalization of primary MEFs.

The 3T3 experiment comparing normoxic and hypoxic conditions, along with the inhibition of *Jarid2* through siRNA, demonstrated that while MEFs under hypoxia reached immortalization, inhibition of Jarid2 slowed down cell growth, suggesting that *Jarid2* inhibition may indeed repress the process of immortalization (Supplementary Fig. S9A). The clonogenicity assay, mimicking stemness properties of cancer cells, further elucidated the role of *Jarid2*. The ability of cells to reconstitute the culture, particularly under hypoxia or with exogenous *Tfcp2l1* expression, was partially suppressed when *Jarid2* was silenced via siRNA (Supplementary Fig. S9B). This implies that *Jarid2* is involved in regulating the stem-like properties induced by hypoxia or *Tfcp2l1* expression. Lastly, the tumorosphere formation assay provided additional evidence supporting the involvement of *Jarid2*. The increase in the number and size of tumorospheres under hypoxic conditions was partially reversed when *Jarid2* was silenced, indicating its contribution to this process ((Supplementary Fig. S9C).

Overall, these findings offer direct experimental support for the proposed role of *Tfcp2l1*-induced gene *Jarid2* in hypoxia-induced immortalization, as outlined in Supplementary Fig. S10. The multifaceted experimental approach strengthens the understanding of the molecular mechanisms underlying cellular immortalization in response to hypoxia and *Tfcp2l1*, emphasizing the significant role of Jarid2 in this process.

## Discussion

Here, we report that the lifespan of MEFs can be increased in hypoxia by activating *Tfcp2l1*, a key regulator of the pluripotency and self-renewal of ESCs. We show that the expression of *Tfcp2l1* is necessary for the proliferation of MEFs and is tightly regulated. In normoxia, when *Tfcp2l1* is overexpressed to levels matching those in hypoxia, genes that regulate proliferation and stemness, such as *Sox2*, *Sox9, Ezh2* and *Jarid2*, are upregulated. Moreover, we show that *Tfcp2l1* can replicate the effect of hypoxia on cellular reprogramming.

The lifespan of primary cells can be extended when they are cultured in hypoxic conditions [8–11]. It has been proposed that hypoxia decreases the production of reactive oxygen species (ROS) and consequent oxidative mechanisms, thus promoting proliferation and suppressing cellular senescence. Parrinello et al. [10] proposed that an adaptive mechanism in hypoxia promotes proliferation despite the high expression of CKIs and p53. Our data agree with the existence of this adaptive response and further suggest that *Tfcp2l1* could be a crucial factor, since its transcriptional activation reduced, either directly or indirectly, the expression of CKIs like *p16*^*INK4a*^, *p15*^*INK4b*^ and *p21*^*Cip1*^, and the transcriptional signaling of p53 induced by proliferative stress in hypoxia.

Experiments using hypoxia, DMOG or the ectopic expression of an oxygen resistant Hif1α mutant suggested that Hif1α can promote the expression of *Tfcp2l1*. We further found active promoters and enhancers containing the Hif1α DNA binding motif close to *Tfcp2l1* in the DNA. These regulatory elements are included in a TAD that defined a potential regulation area that is common between *Tfcp2l1* and another gene relevant for development, *Gli2*. However, this TAD is divided into two sub-TADs, one for each gene, and we found a higher probability for Hif1α DNA binding motifs in the sub-TAD that includes *Tfcp2l1*. These findings point to the specific regulation of *Tfcp2l1* transcription by hypoxia through Hif1α.

As a part of the adaptive response to hypoxia, we found a higher expression of stemness and dedifferentiation genes in hypoxia compared to normoxia. It has been shown that hypoxia promotes pluripotency and self-renewal in ESCs, adult stem cells, iPSCs and CSCs [12–15]. Thus, hypoxia could promote the unlimited self-renewal potential observed in stem cells by similar mechanisms in naïve primary cells. Interestingly, we observed two genes that were overexpressed in cells immortalized by hypoxia, *Rest* and *Tfcpl21*. While *Rest* has pleiotropic functions and regulates several miRNAs with multiple targets along the genome [48, 49], *Tfcp2l1* is well defined as a main regulator of pluripotency and self-renewal in ESCs and can join Oct3/4, Sox2 and Nanog or the demethylating protein Tet2 to bind to enhancers and promoters of *Oct3/4*, *Nanog*, *Esrrb* and *Klf4* [50–52]. Thus, *Tfcp2l1* could be responsible for the adaptive response to hypoxia by increasing the lifespan of MEFs and upregulating the expression of several stemness genes.

When the expression of Tfcp2l1 was downregulated using shRNAs, the cells stopped proliferating and acquired senescence features in hypoxia and also in early passages of normoxia. This observation suggests that *Tfcp2l1* is necessary for the maintenance of proliferation independently of the oxygen condition. Further, the expression of *p16*^*INK4a*^ increased, but we did not observe any change in the expression of stemness genes. This finding implies that that the sudden downregulation of *Tfcp2l1* may activate the expression of CKIs to stop proliferation and initiate senescence despite the high expression of some stemness- genes. However, it is possible that the expression of stemness-associated genes changes at later times. Additionally, hypoxia may have redundant and parallel regulation pathways for stemness genes that function despite the downregulation of *Tfcp2l1*, at least, during our short observation periods.

On the other hand, when the expression of Tfcp2l1 was ectopically upregulated (1000-fold increase in the average mRNA levels observed in hypoxia), MEFs stopped proliferating with senescence features in both normoxia and hypoxia. We observed high levels of CKI expression and an increase in acid β-galactosidase activity along with an increase in the expression of stemness-associated genes. These findings suggest again a dual function of *Tfcp2l1*: regulating stemness properties and proliferation. Additionally, if *Tfcp2l1* regulates proliferation, very high levels of its expression should be sensed as a hyperproliferative stimulus to induce OIS [4, 53]. At the same time, if *Tfcp2l1* regulates the expression of stemness genes like *Oct3/4*, *Sox2*, *Klf4* and *Nanog*, then the phenotype observed with the ectopic expression of *Tfcp2l1* should initiate a cellular response like reprogramming induced senesce (RIS) [54, 55].

Only when the ectopic overexpression of *Tfcp2l1* produced levels comparable to hypoxia did we observe immortalization in normoxia. We achieved this effect using a doxycycline-inducible system. We found stemness genes and Tfcp2l1 pathway genes had increased expressions, but CKI expressions were unchanged. Other pluripotency genes show a similar regulatory mechanism. For example, *Oct3/4* can induce the differentiation of ESCs if its expression is ectopically regulated outside its physiological range [14]. This has been described specifically for *Tfcp2l1* before. It has been shown that only when *Tfcp2l1* ectopic expression induces levels like those reached by the addition of Lif to the culture medium of ESCs can it replicate its effect on pluripotency and self-renewal in these cells [42]. This explains why the extreme upregulation or downregulation of *Tfcp2l1* compromises the lifespan of MEFs.

We found that Tfcp2l1 binds to a bigger number of genes in hypoxia than normoxia, suggesting that hypoxia increases Tfcp2l1 activity as a transcription factor. Indeed, a subset of CREs containing Tfcp2l1 motifs are specific of hypoxia and associate to genes of the transcriptional response to hypoxia. We also found Tfcp2l1 peaks common to both samples, which could suggest a basal activity of Tfcp2l1 that is not enough to immortalize cells in normoxia. Functional enrichment analysis showed that in immortalized hypoxic MEFs, Tfcp2l1 binds to 46 genes that regulate proliferation, including *Il1b*, *Il2*, *Klf10*, *Pdgfra*, *Rgcc*, *Sox2*, *Sox9*, *Sox11*, *Tdgf1* and *Tgfa*. We confirmed by Chip-qPCR the binding of Tfcp2l1 to *Sox2*, *Sox9* and *Tgfa*, which are overexpressed in MEFs cultured in hypoxia. This finding explains the causal role of Tfcp2l1 in the physiological effect of hypoxia. Tfcp2l1 promotes the expression of *Tgfa*, which can activate proliferation pathways in the cell [56]. Furthermore, Tfcp2l1 increases the expression of *Sox2*, part of the pluripotency regulation core [57], and *Sox9*, which participates in stemness regulation when expressed in adult stem cells [58].

Additionally, we found common peaks associated to polycomb repressor complex 2 (PCR2) *Jarid2* and *Ezh2*, which were overexpressed in hypoxia compared to normoxia. Polycomb group of proteins (PcG) form two different repressive complexes, Polycomb repressive complex 1 and 2 (PRC1 and PRC2) [59]. In hypoxia, Tfcp2l1 upregulates the expression of *Sox9* (Fig. 1E and Supplementary Figs. S4 and S5), whose expression is associated with expression of *Bmi1*, a component of PRC1 [60]. Tfcp2l1 also binds DNA regions associated to PRC2 components, *Ezh2* and *Jarid2*, which are overexpressed in hypoxia too (Fig. 5G, H). Together, PRC1 and PRC2, can silence *INK4a/Arf*, allowing proliferation in hypoxia as it has been described before [61]. Our findings suggest that Tfcp2l1 indirectly regulates the *INK4a* locus through the transcriptional regulation of PRC1 and PRC2 (Supplementary Fig. S6).

Our work is centered around understanding the signals that drive the dedifferentiation of mature cancer cells into CSCs, essentially exploring the factors that induce pluripotency—a crucial aspect of reprogramming. Our findings regarding hypoxia/immortalization and the role of Tfcp2l1 in MEFs, along with the mechanistic insight into the role of Jarid2 in reprogramming and its inhibition of the INK4 locus to increase reprogramming, are significant contributions. These discoveries align with prior studies by Li et al. and Banito et al., which demonstrated that deletion of the INK4 locus enhances reprogramming [54, 55].

Our results show that *Tfcp2l1* is responsible for the increase in proliferation of MEFs in hypoxia, which upregulates stemness-associated genes and downregulates CKI expression. We therefore investigated if *Tfcp2l1* could be a molecular factor responsible for the increase of reprogramming in hypoxia. We found that *Tfcp2l1* overexpression replicates the effects of hypoxia on reprogramming and that its downregulation in hypoxia decreases the number of iPSC colonies to a similar number obtained in normoxia. Furthermore, we shown that this effect could be dependent on the Jarid 2 activation by Tfcp2l1 which would maintain repressed INK4 locus, bypassing senescence. This finding is consistent with transcriptional data from iPSCs experiments in normoxia and hypoxia where changes in the expression of stemness and proliferation regulators can be observed as *Tfcp2l1* expression is upregulated during reprogramming

## Conclusions

To conclude, our data suggest that hypoxia increases the lifespan of MEFs through the activation of a single gene, *Tfcp2l1*. This transcription factor can bind the DNA in regions associated to genes that regulate proliferation, such as *Sox2*, *Sox9* and *Tgfa*, whose expression is increased in hypoxia. Furthermore, *Tfcp2l1* is responsible for the increase of reprogramming in hypoxia. As reported in this study, in MEFs, the nuclear core of pluripotency can be activated through the direct regulation of *Sox2* and *Jarid2* by Tfcp2l1 in hypoxia. Thus, MEFs acquire a stemness phenotype, which drives them into immortalization under hypoxic conditions. Further, these results indicate that the activation of *Tfcp2l1* by hypoxia could be relevant in the immortalization prior to malignant transformation, facilitating tumorigenesis. Furthermore, in the tumoral microenvironment, which is hypoxic, *Tfcp2l1* expression could support CSC generation by promoting pluripotency and cellular dedifferentiation. The results obtained from the series of experiments presented in this work collectively support the idea that *Tfcp2l1* plays a critical role in the process of immortalization under hypoxic conditions facilitated by *Jarid2*.

The interplay between the regulatory mechanisms governing cellular differentiation, pluripotency, and the transition to CSCs holds immense potential for understanding cancer progression and potentially devising novel therapeutic strategies.

### Supplementary information

Supplementary Material

aj.checklist

## Data Availability

Dataset for ChIP-seq data (code: GSE173648) was stored at GEO. Reviewer’s token: wrirceoehhgnvop. Additional datasets used in this study are public, references and codes can be consulted in “Methods” description.
