# Supplementary material for: Hypoxia-induced immortalization of primary cells depends on Tfcp2L1 expression
Source: Cell Death Dis. 2024 Feb 28;15(2):177. doi: 10.1038/s41419-024-06567-z (PMC10902313; doi:10.1038/s41419-024-06567-z)

SUPPLEMENTAL FIGURES

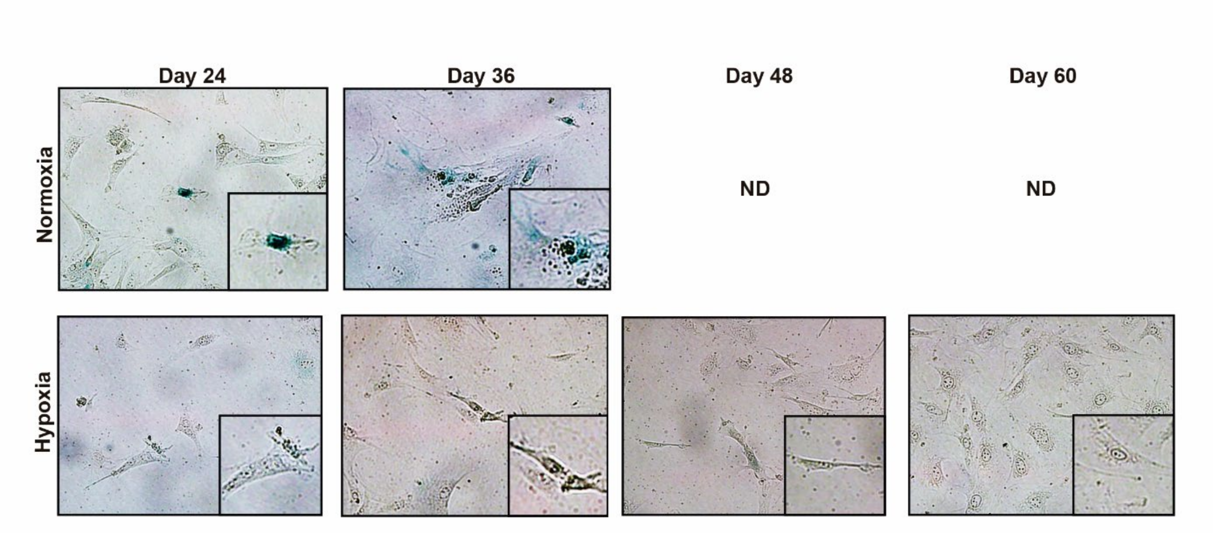

Fig Supp. 1 Cellular morphology and X-Gal staining. The cellular morphology was observed in MEFs used for the acid β-galactosidase activity assay in (Fig1B).

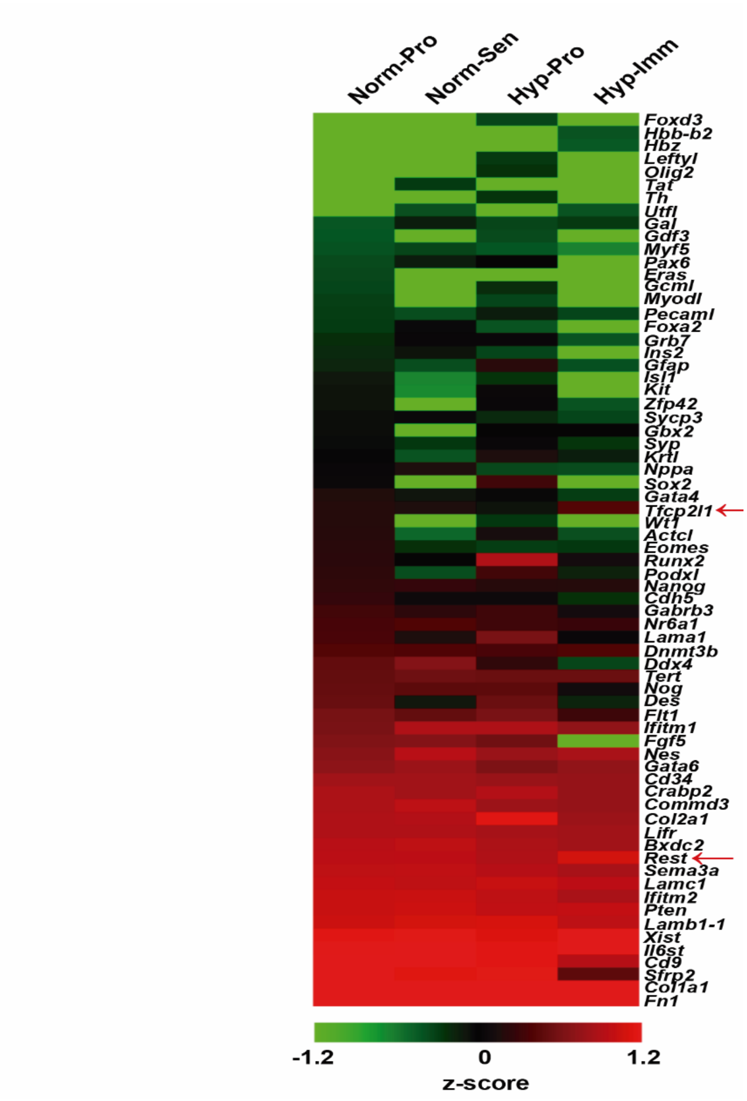

Fig Supp. S2. Differential expression analysis of stemness genes. We used a qPCR array to measure the expression of 96 genes. Results were normalized to the proliferative sample from normoxia, and z-scores were calculated.

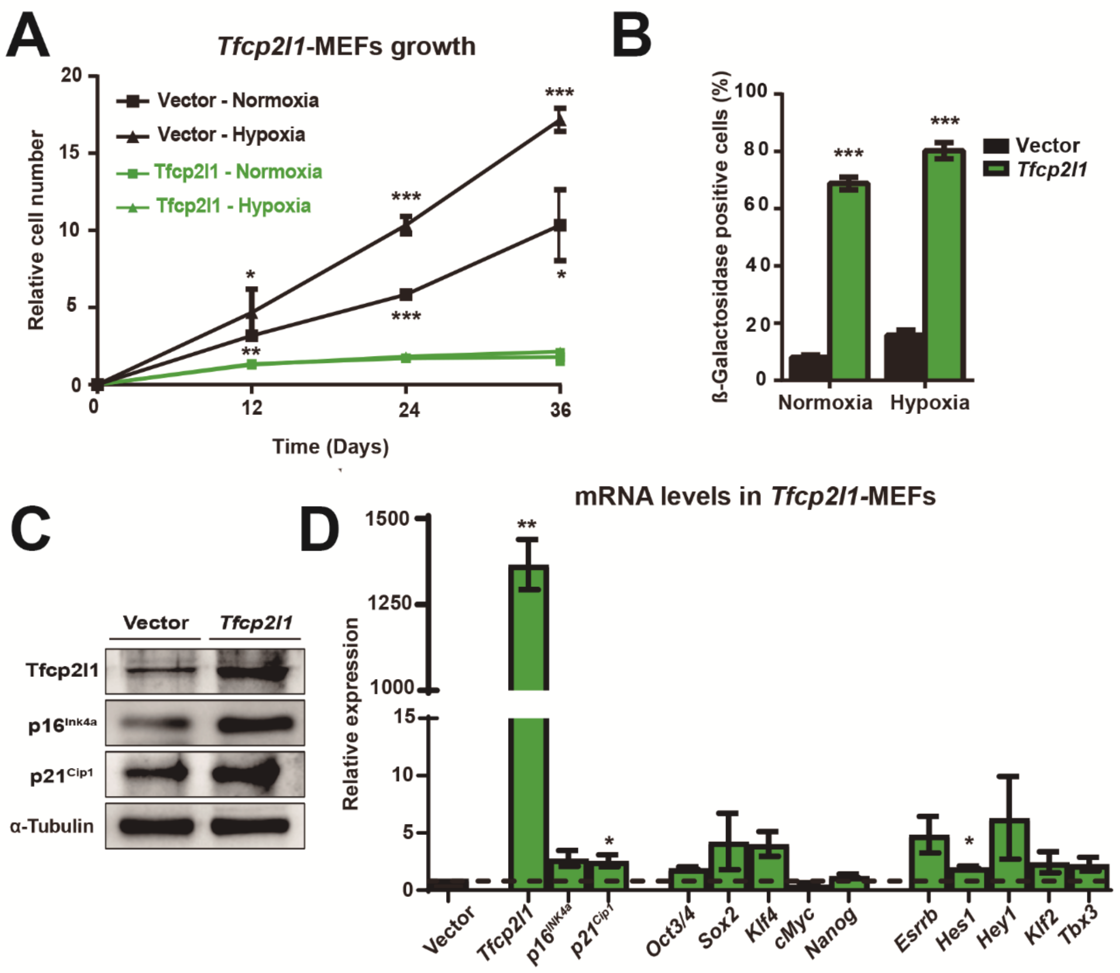

Fig. Supp. S3. Overexpression of high levels of *Tfcp2l1* produces senescence in MEFs, while similar levels to those produced physiologically by hypoxia extend the lifespan. A) Relative cell growth of MEFs*.* MEFs were initially cultured in hypoxia or normoxia, transduced with a control vector or *Tfcp2l1* cDNA, and after selection, cultured in the 3T3 modified protocol. **B) β-galactosidase activity.** The transduced and selected MEFs were fixed after 3 days of culture in the 3T3 modified protocol and stained with X-Gal. **C) Tfcp2l1 and CKIs protein levels.** The protein levels of Tfcp2l1, $p16^{INK4a}$ and $p21^{Cip1}$ were analyzed after 3 days in the 3T3 modified protocol. **D) mRNA levels of *Tfcp2l1,* CKIs, stemness-associated genes and genes from the Tfcp2l1 pathway in development.** mRNA levels of *Tfcp2l1*, $p16^{INK4a}$, $p21^{Cip1}$, *Oct3/4, Sox2, Klf4, cMyc, Nanog, Esrrb, Hes1, Hey1, Klf2* and *Tbx3* were analyzed by qPCR after 3 days in the 3T3 modified protocol.

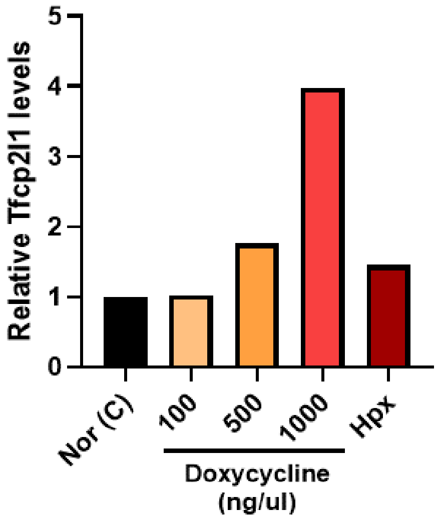

Fig Supp. S4. Quantification of hypoxia-induced Tfcp2l1 protein levels.

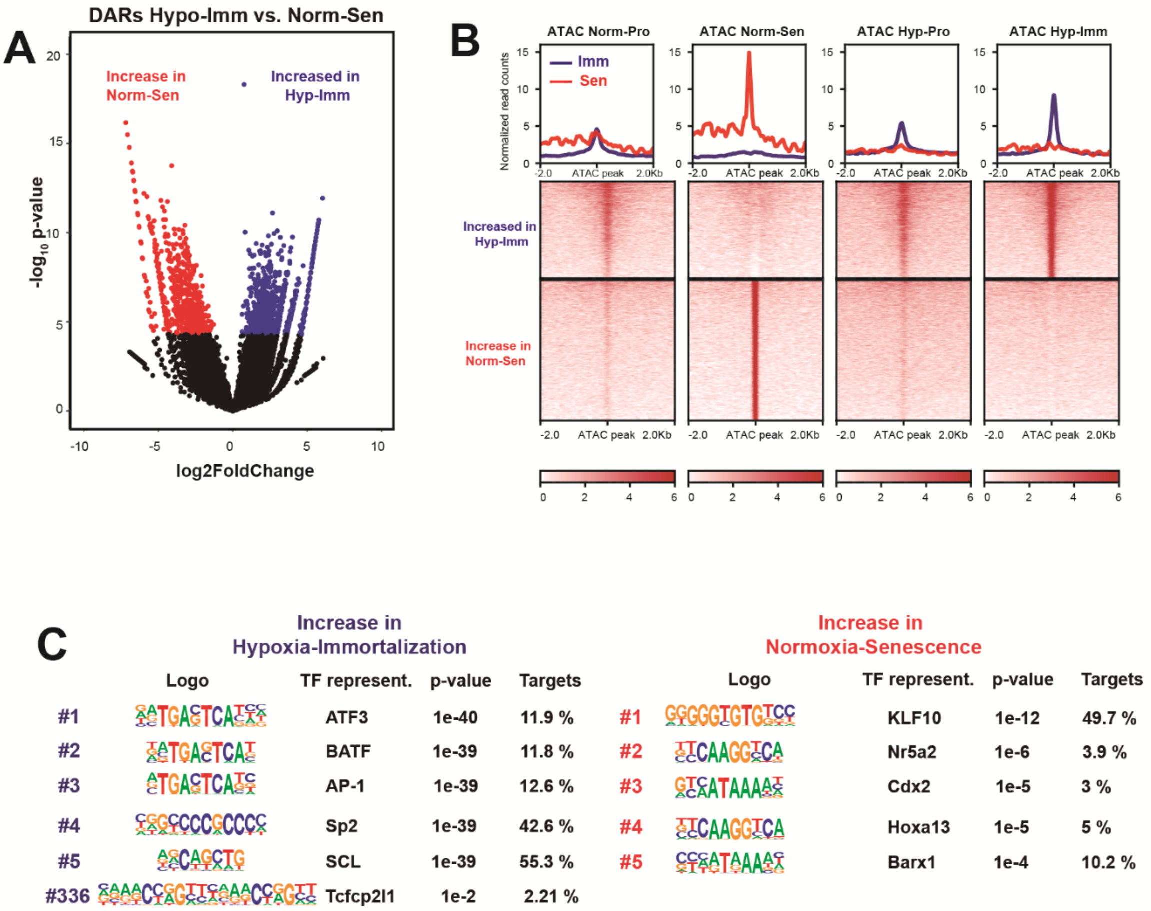

Fig. Supp. S5. Differential accessibility analyses in senescent vs immortalized MEFs. A) Volcano plot showing the -$\log_{10}$-p-value versus the $\log_2$-fold-change of ATAC-seq peaks. Peaks showing a statistically significant change (P value < 0.05) are highlighted in blue (increased peaks in immortalized MEFs) or red (increased peaks in senescent MEFs). **B)** Heatmaps plotting normalized ATAC-seq signals at differentially accessible regions (DARs) from (A) in proliferating MEFs in normoxia (Norm-Pro), senescent MEFs (Norm-Sen), proliferating MEFs in hypoxia (Hyp-Pro) and immortalized MEFs (Hyp-Imm). **C)** Motif enrichment analyses of the ATAC peaks increased in immortalized (left) and senescent MEFs (right). The five motifs with the lowest p values are shown in each case, as well as the position of the Tcfcp2l1 motif when identified as significantly enriched.

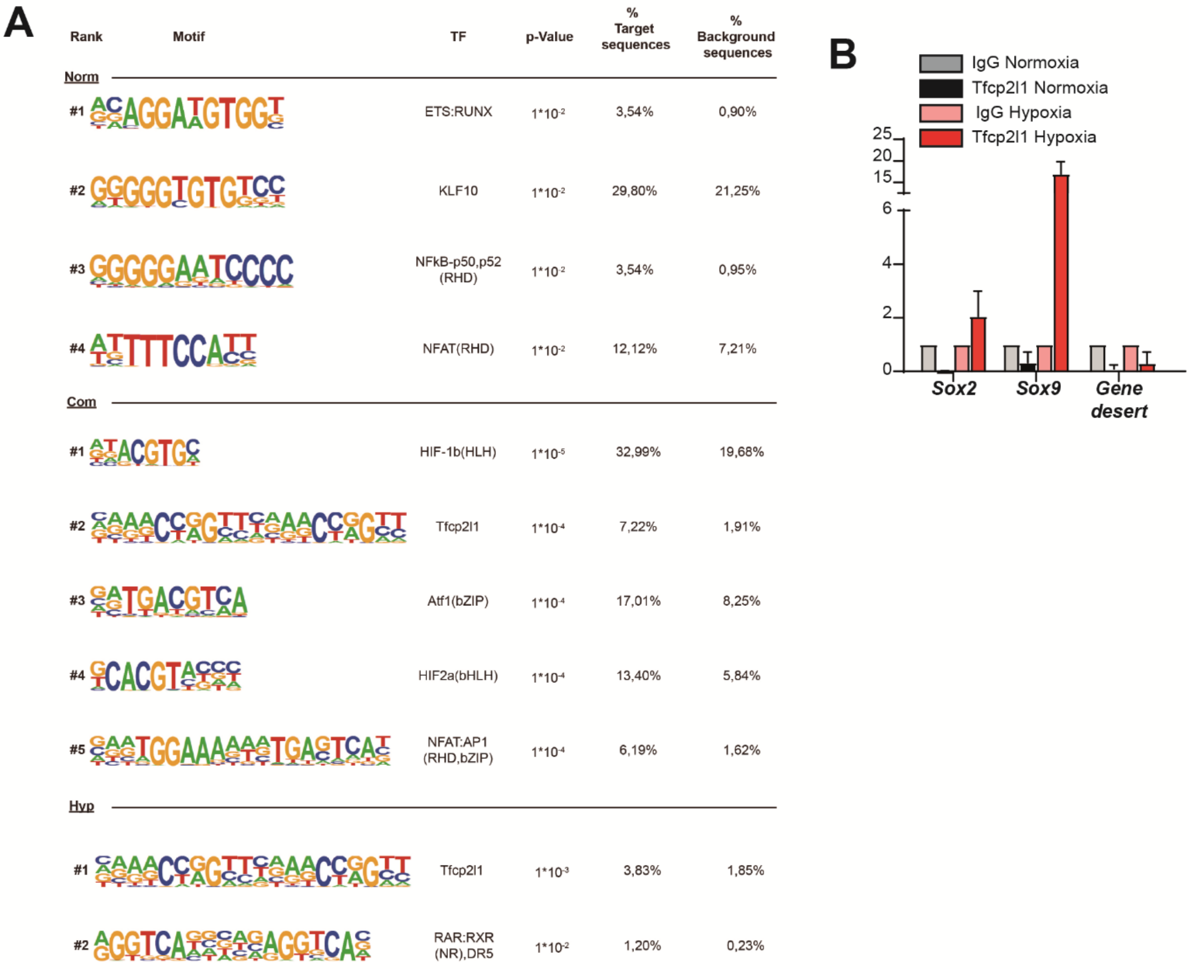

Fig. Supp. S6. Binding of Tfcp2l1 to chromatin. A) Motif enrichment analysis in the 3 groups of peaks found in the ChIP-Seq experiments. Most significant enrichments are shown according to their p-values. The percentage of target sequences (peaks) and unspecific sequences (background) where the motif was found is also shown. **B)** Validation of Tfcp2l1 chromatin binding by ChIP-qPCR in genomic regions associated to *Sox2*, *Sox9* and *Tgfa*. Negative controls used were immunoprecipitation of IgG, non-coding region Gene Desert; positive controls were anti-RNA polymerase II and primers against the first exon of α-tubulin. Ct data were analyzed using the relative quantification method.

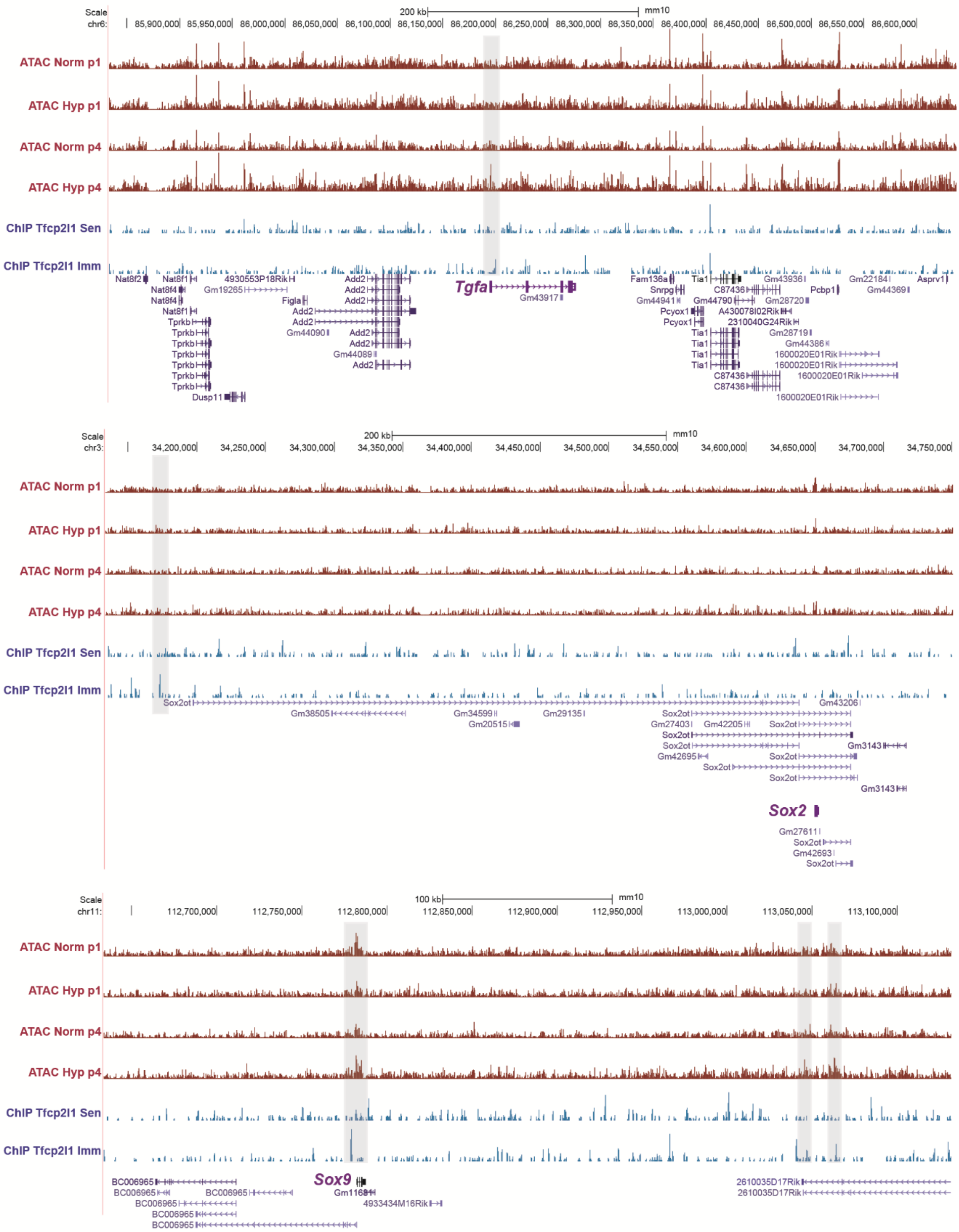

Fig. Supp. S7. Genomic tracks showing ATAC-seq and ChIP-seq signals in the analyzed conditions for the *Tgfa, Sox2* and *Sox9* gene loci. TF, transcription factor.

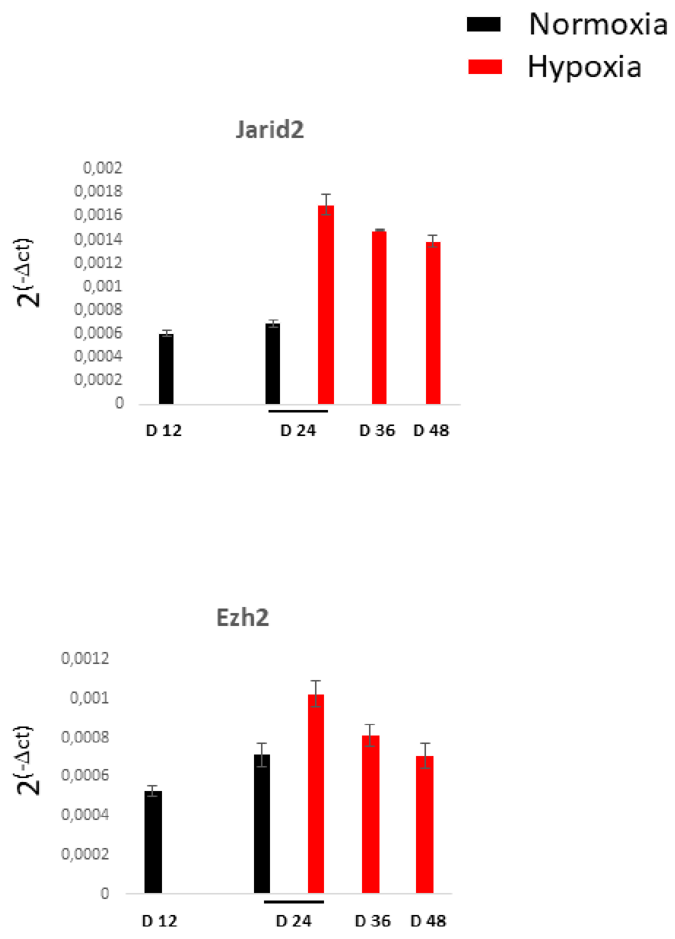

Fig. supp. S8. Q-RT-PCR measurement of the transcription levels of activation of Tfcp2l1-target genes linked to stemness, Jarid2, and Ezh2 upon hypoxia activation during immortalization.

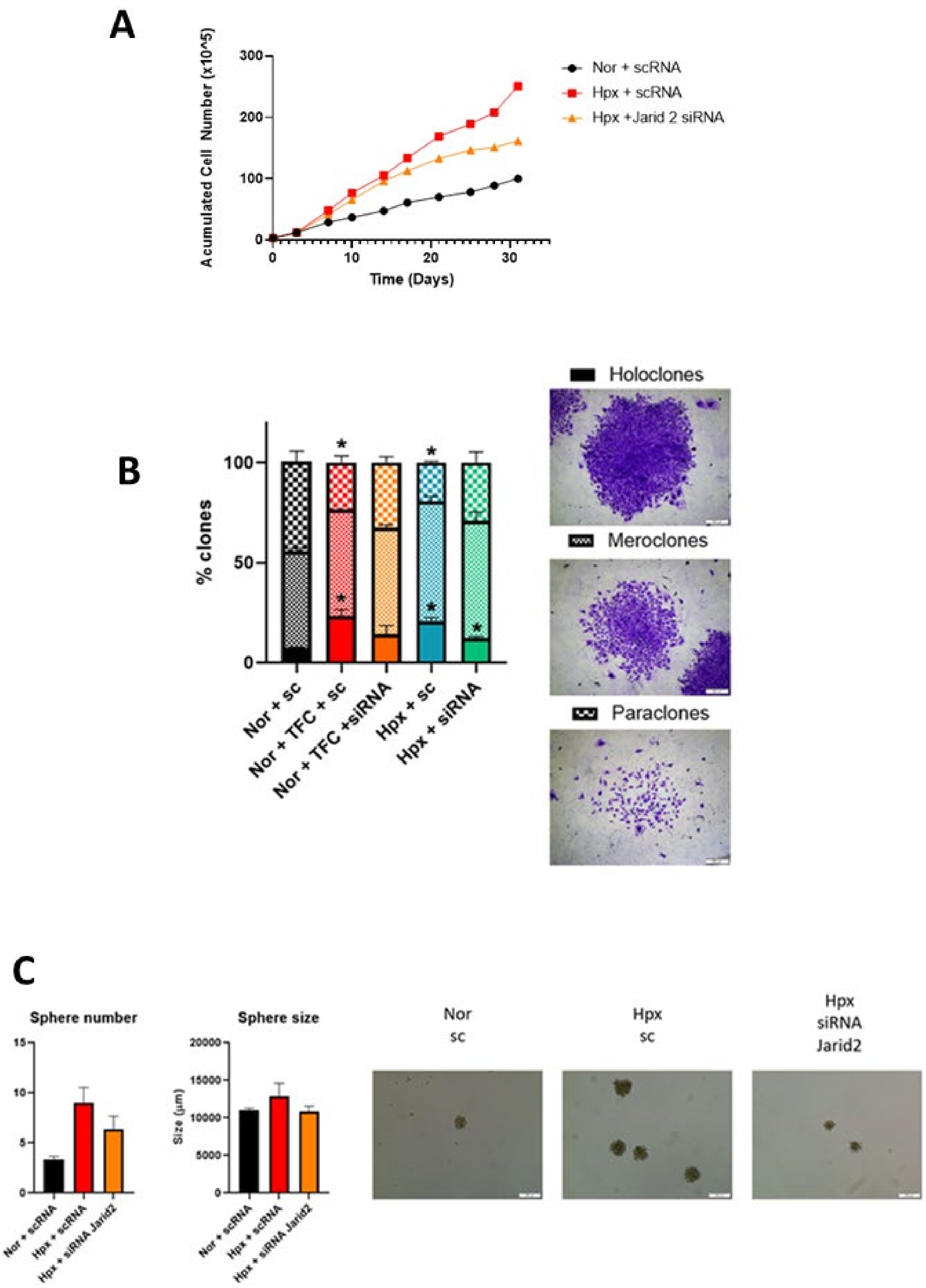

Fig. supp. S9. **A)** The 3T3 experiment comparing normoxic and hypoxic conditions, along with the inhibition of *Jarid2* through siRNA. Legend: Nor+ScRNA:Normoxia+Scramble siRNA; Hpx+ScRNA: Hypoxia+Scramble siRNA; Hpx+Jarid2siRNA: Hypoxia+SiRNA for Jarid2 gene. **B)** The clonogenicity assay, mimicking stemness properties of cancer cells, comparing normoxic and hypoxic

conditions, along with the inhibition of *Jarid2* through siRNA. Legend: Nor+Sc:Normoxia+Scramble siRNA; Nor+TFC+SC: Normoxia+overexpression of TFC+ Scramble siRNA; Nor+TFC+siRNA: Normoxia+overexpression of TFC+ Jarid2 siRNA; Hpx+Sc: Hypoxia+Scramble siRNA; Hpx+siRNA: Hypoxia+SiRNA for Jarid2 gene. **C)** Tumorosphere formation assay provided additional evidence supporting the involvement of *Jarid2*. Legend: Nor+ScRNA:Normoxia+Scramble siRNA; Hpx+ScRNA: Hypoxia+Scramble RNA; Hpx+Jarid2siRNA: Hypoxia+SiRNA for Jarid2 gene.

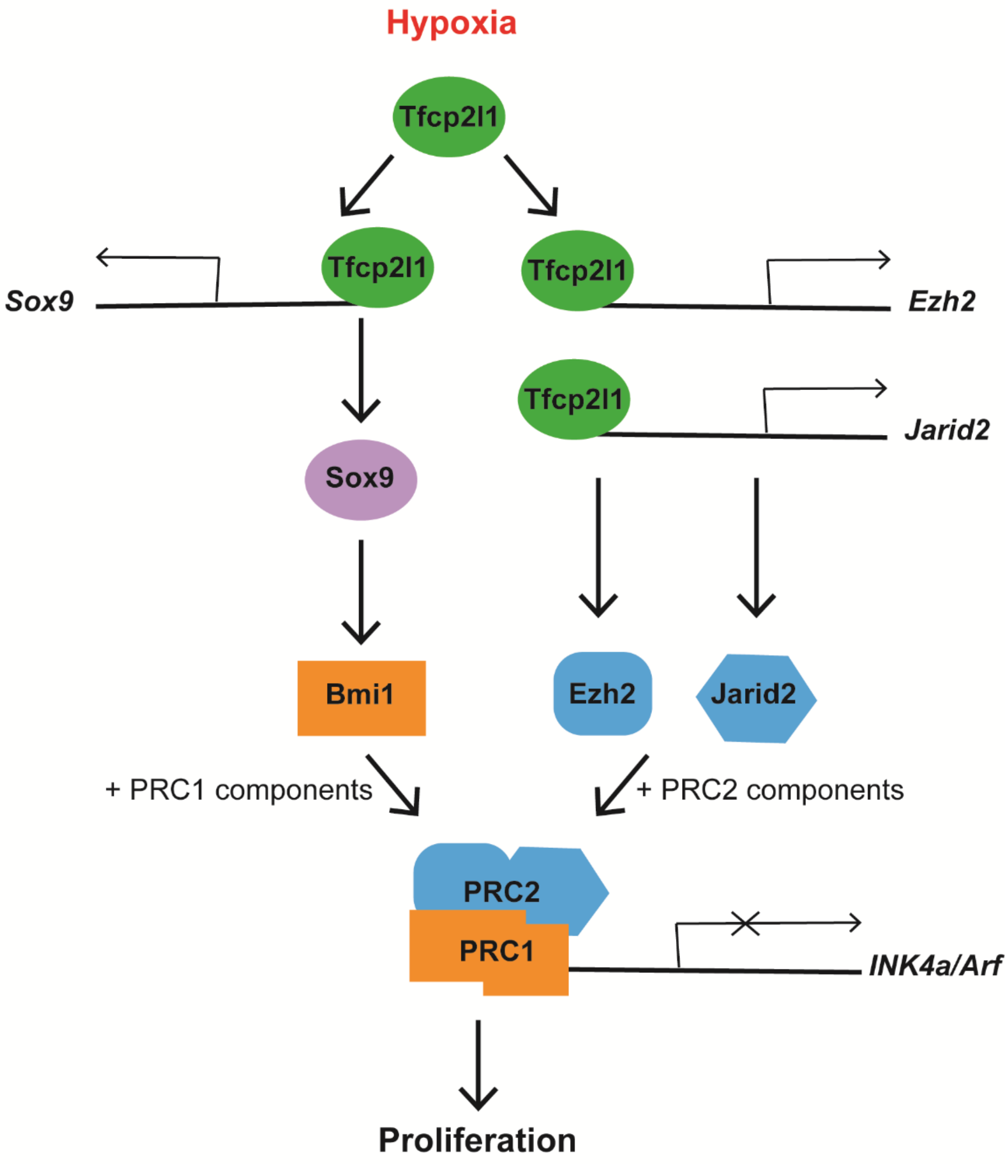

Fig. Supp. S10. Suggested model for Tfcp2l1 modulation of *Ink4a/Arf* locus in hypoxia. In hypoxia, Tfcp2l1 upregulates the expression of Sox9, whose expression is associated with expression of Bmi1, a component of PRC1. Tfcp2l1 also binds DNA regions associated to PRC2 components, Ezh2 and Jarid2, which are overexpressed in hypoxia too. Together, PRC1 and PRC2, can silence *INK4a/Arf*, allowing proliferation in hypoxia.

FIGURE 1.D

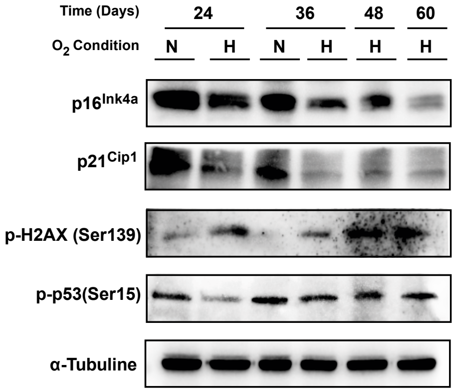

p16

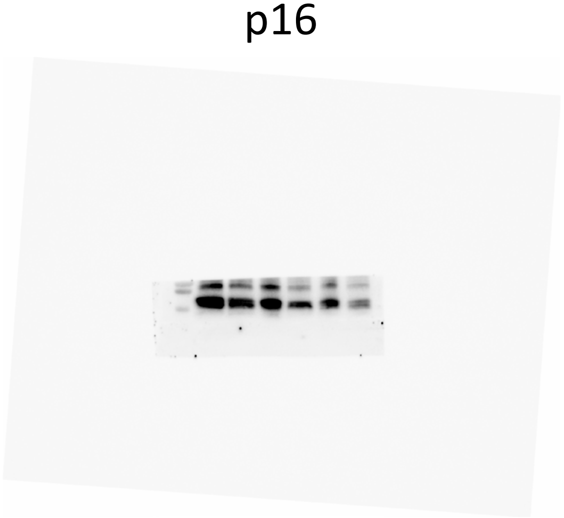

p21

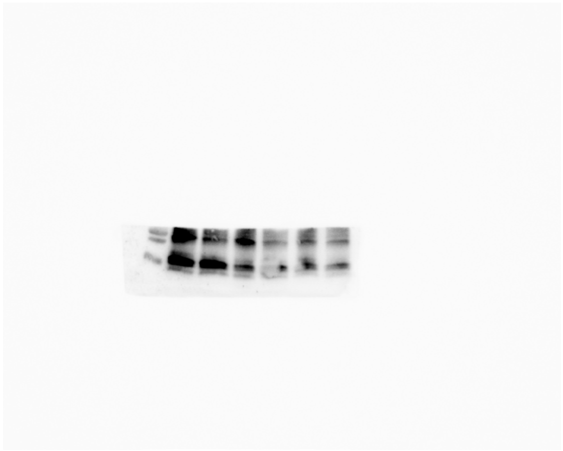

p-p53(Ser15)

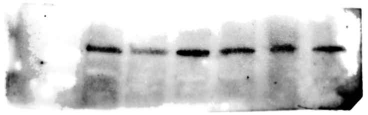

P-H2AX(Ser139)

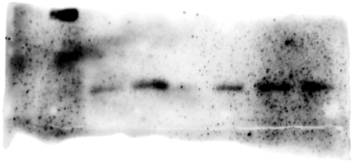

a-Tubuline

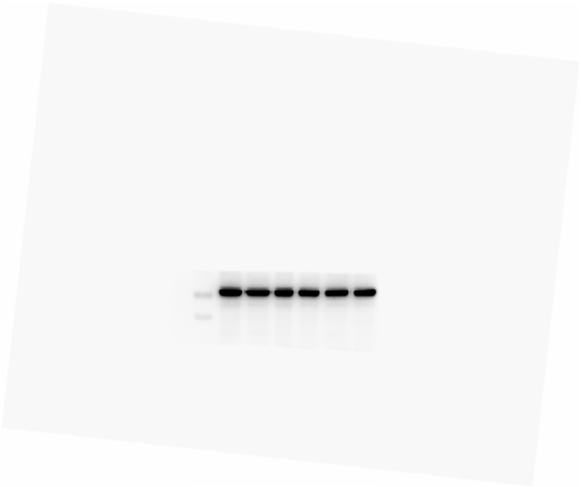

FIGURE 1.F

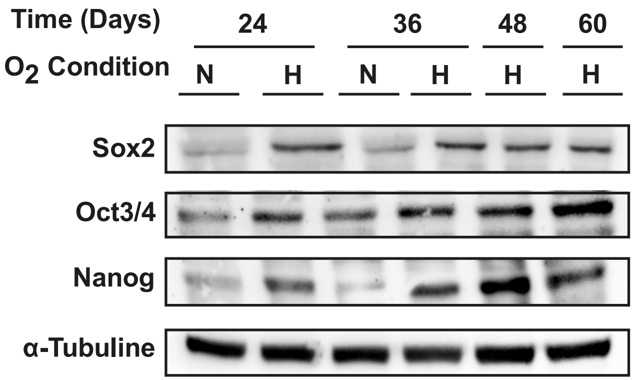

Sox2

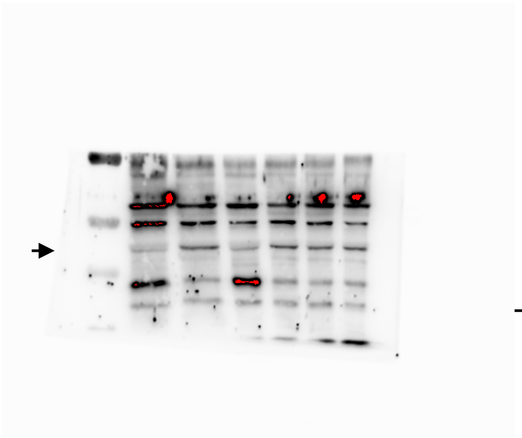

Oct3/4

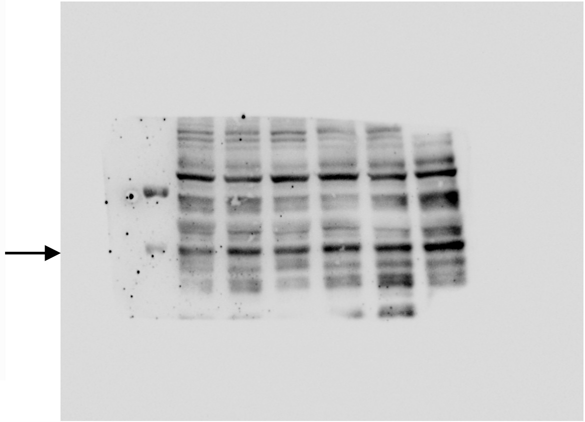

Nanog

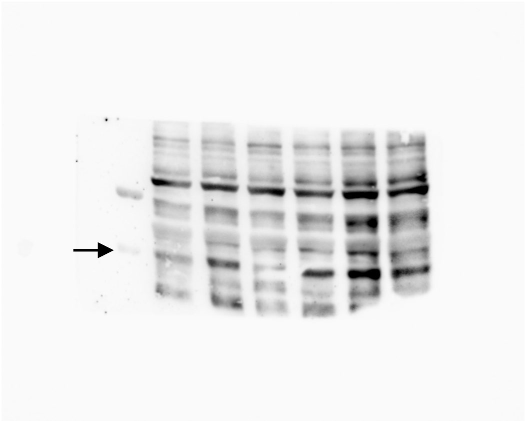

a-Tubuline

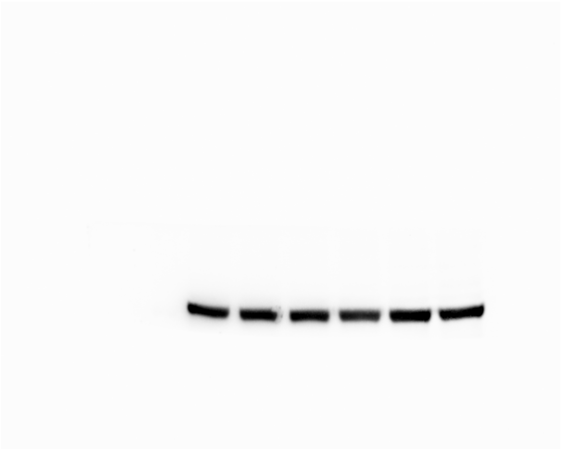

FIGURE 1.H

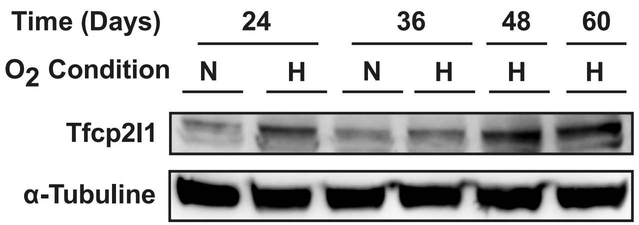

Tfcp2l1

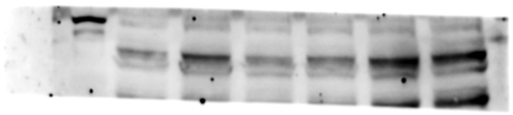

a-Tubuline

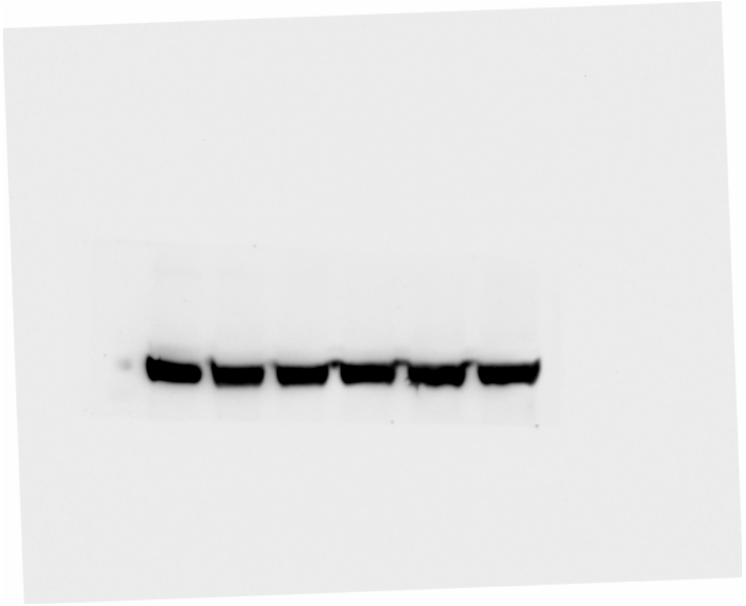

FIGURE 2.G

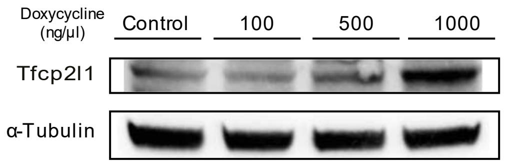

Tfcp2l1

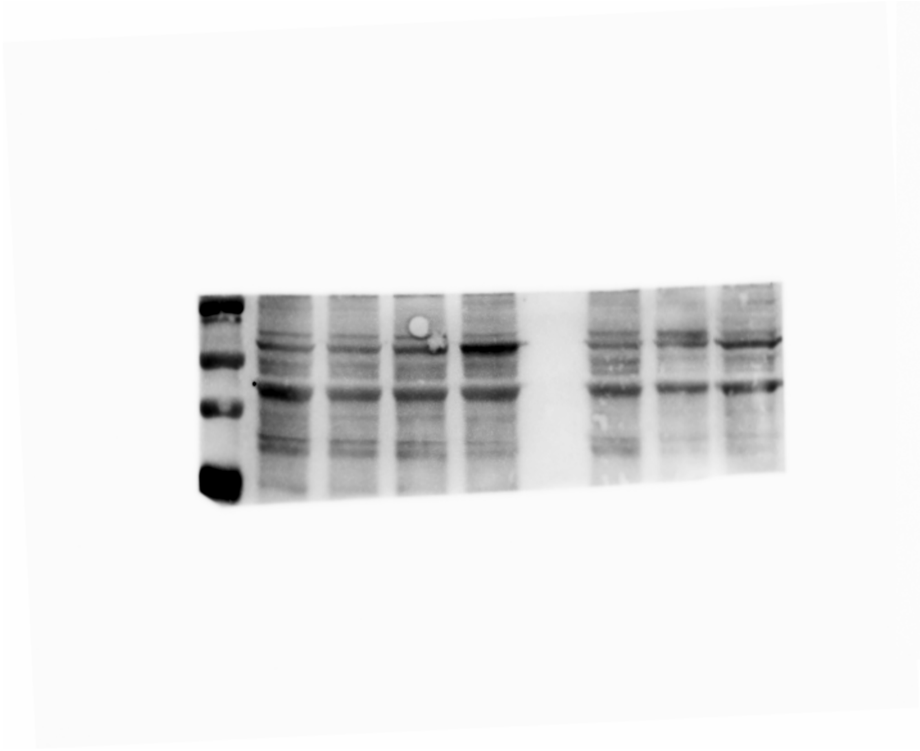

a-Tubuline

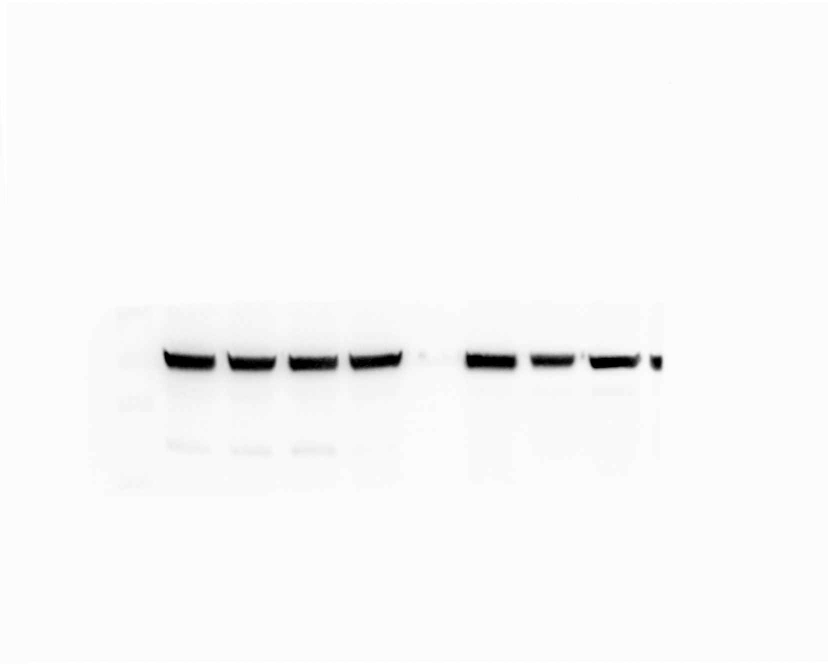

FIGURE 3.A

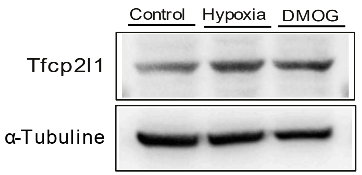

Tfcp2l1

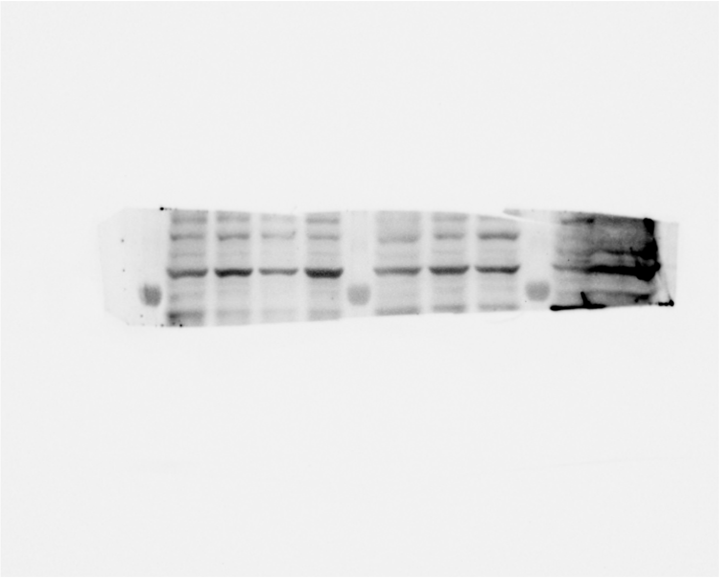

a-Tubuline

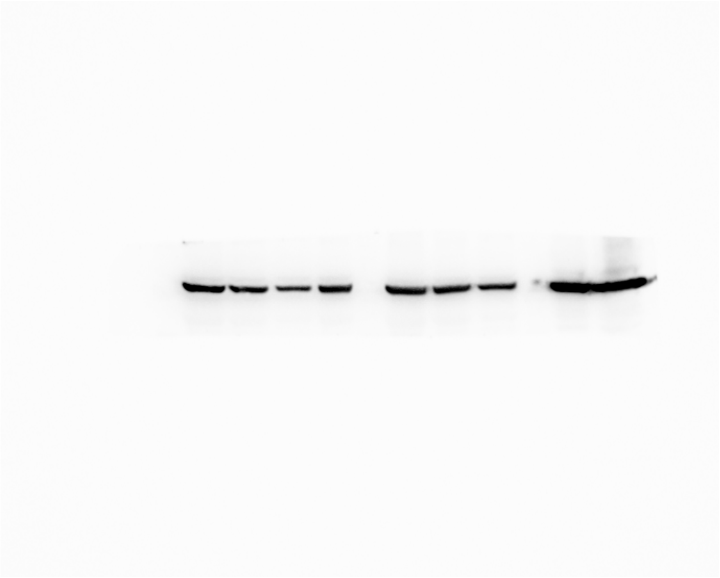

FIGURE 3.D

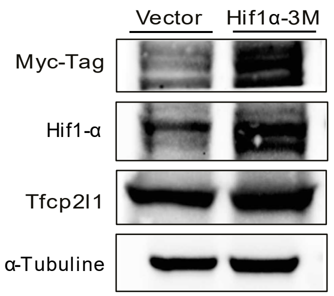

Myc-Tag

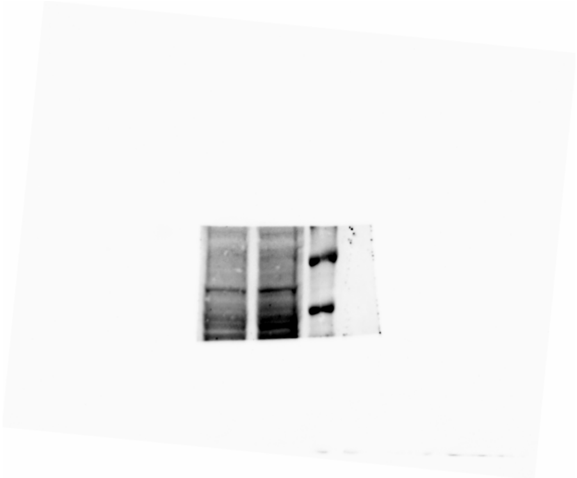

Hif1-a

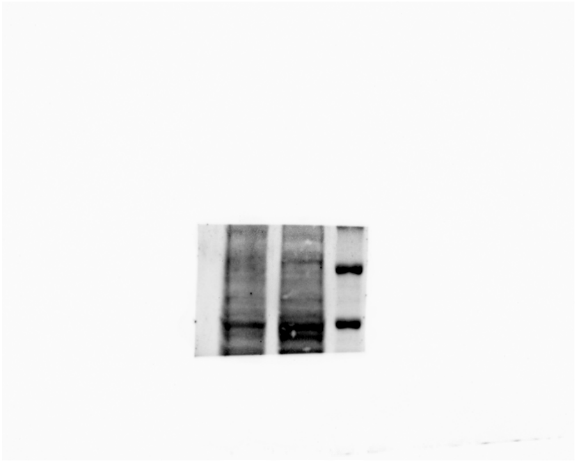

Tfcp2l1

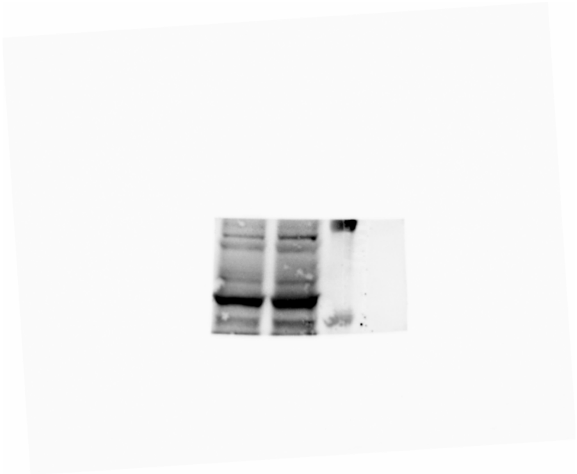

a-Tubuline

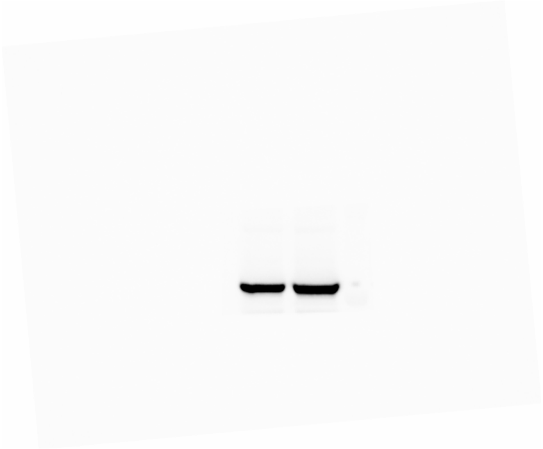

FIGURE S3.C

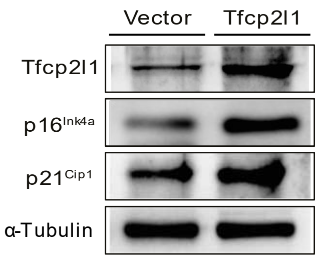

Tfcp2l1

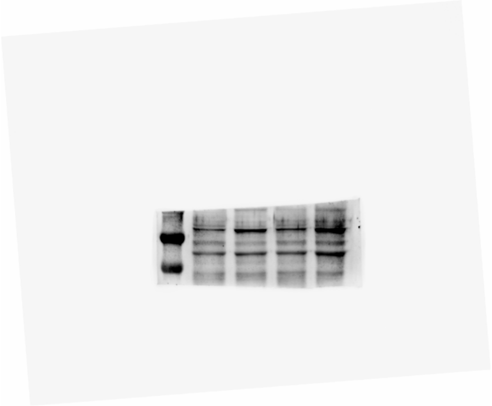

p16

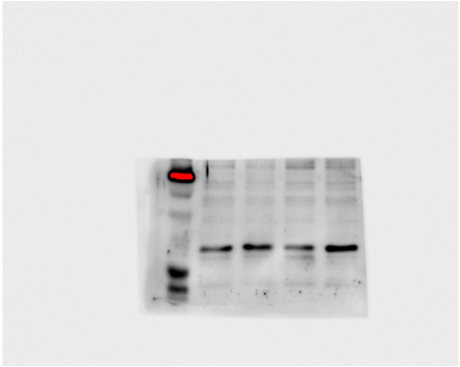

p21

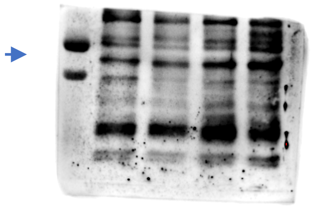

a-Tubuline

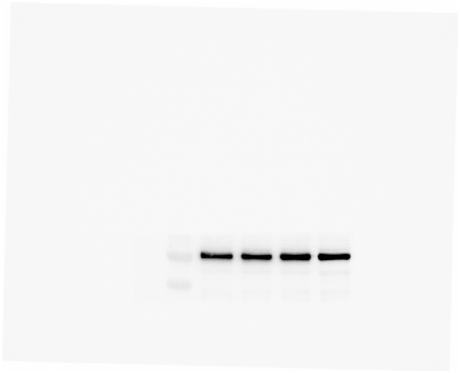

Supplement: Supplementary file 1 — Supplementary Material [file 41419_2024_6567_MOESM1_ESM.pdf]
